# Assessing the Impact of U.S. Food Assistance Delivery Policies on Child Mortality in Northern Kenya

**DOI:** 10.1371/journal.pone.0168432

**Published:** 2016-12-20

**Authors:** Alex Nikulkov, Christopher B. Barrett, Andrew G. Mude, Lawrence M. Wein

**Affiliations:** 1 Graduate School of Business, Stanford University, Stanford, California, Unites States of America; 2 Charles H. Dyson School of Applied Economics and Management, Cornell University, Ithaca, New York, United States of America; 3 International Livestock Research Institute, Nairobi, Kenya; The Hospital for Sick Children, CANADA

## Abstract

The U.S. is the main country in the world that delivers its food assistance primarily via transoceanic shipments of commodity-based in-kind food. This approach is costlier and less timely than cash-based assistance, which includes cash transfers, food vouchers, and local and regional procurement, where food is bought in or nearby the recipient country. The U.S.’s approach is exacerbated by a requirement that half of its transoceanic food shipments need to be sent on U.S.-flag vessels. We estimate the effect of these U.S. food assistance distribution policies on child mortality in northern Kenya by formulating and optimizing a supply chain model. In our model, monthly orders of transoceanic shipments and cash-based interventions are chosen to minimize child mortality subject to an annual budget constraint and to policy constraints on the allowable proportions of cash-based interventions and non-US-flag shipments. By varying the restrictiveness of these policy constraints, we assess the impact of possible changes in U.S. food aid policies on child mortality. The model includes an existing regression model that uses household survey data and geospatial data to forecast the mean mid-upper-arm circumference Z scores among children in a community, and allows food assistance to increase Z scores, and Z scores to influence mortality rates. We find that cash-based interventions are a much more powerful policy lever than the U.S.-flag vessel requirement: switching to cash-based interventions reduces child mortality from 4.4% to 3.7% (a 16.2% relative reduction) in our model, whereas eliminating the U.S.-flag vessel restriction without increasing the use of cash-based interventions generates a relative reduction in child mortality of only 1.1%. The great majority of the gains achieved by cash-based interventions are due to their reduced cost, not their reduced delivery lead times; i.e., the reduction of shipping expenses allows for more food to be delivered, which reduces child mortality.

## Introduction

Undernutrition causes ≈45% of deaths in children under 5 years old [1], and the supply of international food assistance is much less than the demand [2]. Although the U.S. is the world’s largest provider of food assistance [3] and recently increased its use of cash-based food assistance [4], it is one of the few countries (along with Brazil and China, although they provide much less food aid than the U.S. [5]) that provides the majority of its assistance via transoceanic shipments of commodity-based in-kind food aid [2]. Other countries rely on local and regional procurement (LRP), where food is bought in the recipient country (local) or a neighboring country (regional) and then delivered to the target recipients [6], and direct delivery of cash or vouchers to the target recipients [7]. LRP provides more timely (62% delay reduction) and less costly (>50% reduction) delivery than transoceanic shipments [8]. Although the cost and timeliness of cash and vouchers depend on the complexity of the distribution [9], they are thought to be much more similar to LRP’s cost and timeliness than to those of transoceanic shipments. In addition, transoceanic shipments of in-kind food aid can cause volatility in local markets where monetization (i.e., the food is sold and the money is used for development programs) is used, and key trade partners view it as a form of export subsidy that violates the intent of international trade agreements [10].

The presidential administrations of George W. Bush and Barack Obama have sought, with limited success, greater flexibility in the use of food assistance funding [10]. The Agricultural Act of 2014 (Public Law, or P.L., 113-79, known as the 2014 farm bill) has authorized LRP for $80M/yr through fiscal year 2018 for use in emergency food assistance [11] (although funding has yet to be appropriated), which is to be managed by the U.S. Department of Agriculture. In contrast, the Food for Peace Act (P.L. 480) Title II program, which is managed by the U.S. Agency for International Development (USAID) and represents ≈80% of the U.S. food assistance budget [11], is required to use transoceanic food shipments.

Not all of the commodity shipments originating from the P.L. 480 Title II program are actually distributed to malnourished people. Until recent years, much of it was sold to commercial buyers immediately upon arrival in the destination country, with the resulting funds used for multi-year projects that address the causes of undernutrition in the targeted locations [11]. These monetized transfers are not simply a slow, high-cost way to deliver food to undernutritioned children, but are a policy tool at the intersection of U.S. domestic agricultural policy, international trade policy, and development policy, which can support poverty reduction and nutrition improvement while attempting to minimize collateral damage (e.g., only displacing other exporters’ shipments to the target countries). However, following the 2014 farm bill, U.S. emergency Title II food aid monetization in sub-Saharan Africa has virtually ended and the cash-based Emergency Food Security Program and Community Development Fund operated by USAID have become a major part of emergency response. Although the development (i.e., non-emergency) Title II programs and other, small programs (e.g., Food for Progress) are heavily monetized, they are far smaller than the emergency programs: in fact, emergency food aid (including Title II emergency food aid, Emergency Food Security Program, Community Development Fund and Bill Emerson Humanitarian Trust) represents 89.5% of total U.S. food assistance [12].

More generally, commodity transfers are just one part of a larger system that includes U.S. cash for LRP, as well as coordination and competition with other donors and other programs that target malnutrition in different ways at different locations. While a hypothetical political-economy model of the U.S. food aid system as a whole would need to take into account the interactions between these programs, and between the U.S. and other food aid donors (i.e., in terms of the assistance modalities used by each player), as well as the legislative and lobbying constraints under which policymakers operate, such an analysis is well beyond the scope of our paper. Rather, we focus on the direct child mortality effects resulting from the portion of U.S. food assistance that is actually used to directly feed undernutritioned children. That is, our model includes only the non-monetized proportion (89.5% overall, but nearly 100% in sub-Saharan Africa) of P.L. 480 aid, which in turn is only a fraction (80%) of what the U.S. and other donors call “food aid.”

In addition to the constraint on transoceanic food shipments, the Cargo Preference Act (P.L. 83-644) requires that 50% of U.S. food aid volume be delivered on U.S.-flag vessels, which increases shipping costs [13] and food insecurity [14]. A reduction from 75% to 50% occurred as a result of the 2012 Surface Transportation Reauthorization Act; in addition, Section 318 of the original version of the 2014 Coast Guard and Maritime Transportation Act of 2014, H.R. 4005, had a provision to revert back to 75% that passed the U.S. House of Representatives on a voice vote, but was not included in the final bill [10].

In this study, we use mathematical modeling and optimization to estimate the impact that restrictions on U.S.’s cash-based interventions (in our model, we conservatively assume that all cash-based interventions have LRP’s cost and timeliness characteristics) and cargo preference have on child mortality in sub-Saharan Africa, which incurred 49.3% of worldwide under-five mortality in 2011 (Table 1 in [15]); i.e., we move beyond the comparison of cost and timeliness [8] and consider child mortality. Because a potential drawback of transoceanic shipments is their lack of responsiveness to changes in food assistance demand, it is important to incorporate forecasts of food assistance demand in our analysis. Our starting point is the model in [16], which uses household survey data and geospatial data to forecast (one and three months into the future) the mean mid-upper arm circumference Z (i.e., standardized from the 1978 CDC/WHO growth charts) value (MUAC-Z) among children less than five years old in the arid region of northern Kenya. We use this model, which is geared at predicting slow-onset emergencies, to forecast mean MUAC-Z in a community for up to nine months in the future. We then develop a generalization of the martingale model of forecast evolution (MMFE) [17], which models the forecast updates as they evolve over time and quantifies the deterioration of the forecasts as they attempt to predict the mean MUAC-Z values further into the future. This MMFE model is embedded into a global inventory management optimization problem as seen from the perspective of the United Nations World Food Program (WFP), which distributes the great majority of the world’s food assistance. In this optimization problem, monthly food assistance deliveries (food increases MUAC-Z values, which reduces mortality) of cash-based interventions and transoceanic shipments are chosen to minimize child mortality subject to an annual budget constraint and to restrictions on the use of cash-based interventions and cargo preference. By varying the restrictiveness of the constraints on cash-based interventions and cargo preference, we estimate the impact that changes in U.S. food assistance distribution policies would have on child mortality.

## Methods

**Fig 1:**
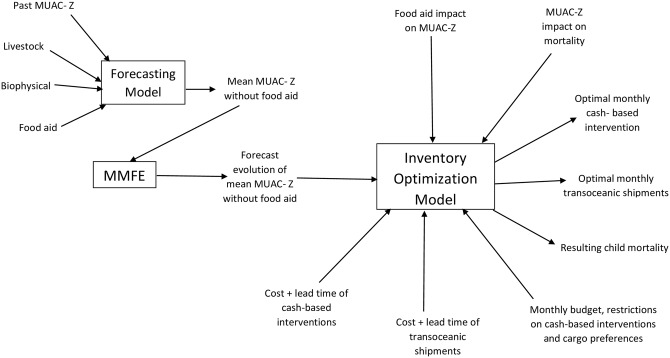
The interrelationships among the components of the model.

All details related to the formulation, calibration and solution of the model appear in the Supporting Material (S1 File). The various components of the analysis are described below, their interrelationships are depicted in Fig 1, and a list of parameters and their values appears in Table 1.

**Table 1:** Parameter values.

Parameter	Description	Value	Reference
*N*	number of children	2236	[16]
*T*	time horizon of model	120 mo	
*H*	forecast horizon	9 mo	§A.3 of the S1 File
${\tilde{\mu }}_{t}$	long-term MUAC-Z forecast for month *t*	Fig B in S1 File	[16]
*σ*	standard deviation of MUAC-Z	0.62	[16], §B.2 of S1 File
Σ^1^, Σ^2^	covariance matrices in MMFE	Figs D, E in S1 File	[16]
*a*, *b*	monthly mortality rate = *e*^*a*−*bz*^	-7.13, 0.722	[18]
*d*	mean increase of MUAC-Z from food aid	0.015/kg	[19], §C.3 of S1 File
*L*_*f*_	lead time of cash-based interventions	3 mo	[8]
*L*_*s*_	lead time of transoceanic shipment	6 mo	[8]
*p*	proportion of shipments on non-US carriers	0.25–1.0	P.L. 83-644
*l*	proportion of WFP’s food via cash-based interventions	0.65–1.0	[6]
*c*_*f*_	cost of cash-based interventions	$0.363/kg	[8]
*c*_*s*_	cost of transoceanic food aid	$0.819−$0.096*p*/kg	[8]
*B*	annual budget for food assistance	$19,867	[8, 16]

### Forecasting Model

We use monthly data from February 2001 to May 2005 in 42 communities in northern Kenya (§A.1 of the S1 File), which includes community-level probability distributions of individual MUAC-Z scores for *N* = 2236 children and community-level data pertaining to livestock, food aid and biophysical variables [16]. Because we will be using the MUAC-Z forecasts to optimize the food deliveries, we modify the MUAC-Z data so that the forecasts are calibrated to forecast the mean MUAC-Z in the absence of the food assistance (this effect is quantified in the next paragraph) that was actually provided. To derive this net MUAC-Z value, for each observation we find the amount of food assistance delivered to the community in each of the last 54 months and then subtract the effect of each portion of this food assistance from the observed realization of MUAC-Z (see §A.2 of the S1 File for details). The dependent variable in the linear regression model (§A.3 of S1 File) is the mean MUAC-Z in the absence of food assistance among children in community *i* in month *t* + *τ* as predicted in month *t*, and the 119 explanatory variables include time-lagged values of MUAC-Z moments, livestock variables, food aid variables and biophysical variables (Table B in S1 File), as well as a community-specific effect and seasonality (i.e., month of the year). When estimating the regression parameters, we start generating forecasts in February 2003 so that each forecast is based on at least two years of data. The empirical relationship between the root mean square error and *τ* leads us to choose a forecast horizon of nine months (Fig A in S1 File). We aggregate forecasts across the 42 communities using a population-weighted average, and our resulting forecast in month *t* for the mean MUAC-Z in the absence of food assistance in month *t* + *τ* in northern Kenya is denoted by *f*_*t*,*t* + *τ*_.

### The Effect of Food Assistance on MUAC-Z

Because factors beyond food, such as disease, affect undernutrition [18], it is important to use results from randomized controlled feeding trials. Consequently, to estimate the effect of food assistance, we use the results from the only large randomized controlled trial with a nontreatment control group that measures the impact on weight-for-height Z scores (WHZ) and height-for-age Z scores (HAZ) [19], where 500 kcal/day administered for three months to children ages 6-60 months led to a persistent (after a six-month follow up) mean WHZ increase of 0.19. Because WHZ and MUAC-Z both measure how thin a child is, we extrapolate the results from WHZ to MUAC-Z. Moreover, as in [20], for lack of data we assume that the mean treatment effect is linear in the amount of calories consumed. Hence, we assume that each kcal/day given for one month causes an increase in the mean MUAC-Z by 0.19/3(500) = 1.3×10^−4^. In addition, the data in [16] measure the amount of food assistance in kg, and we assume that 1 kg of food assistance contains 3679 kcal (§A.2 of S1 File), so that (assuming 30 days per month) 1 kg of food assistance increases MUAC-Z by *d* = 3679(1.3×10^−4^)/30 = 0.015.

### Forecast Evolution Model

The forecast evolution model is not a forecasting model, but rather is a model of the evolution of a forecasting system; although in our case the forecasts are generated by the regression model described earlier, a forecast evolution model can be applied to any set of forecasts, e.g., one generated by a human expert without the aid of a statistical model.

Our inventory optimization model requires us to understand how the forecast for the mean MUAC-Z in month *t* changes as we approach month *t*, which is precisely what the forecast evolution model does. The evolution of the forecasting model is specified by the forecast updates, *ϵ*_*t*−*τ*,*t*_ = *f*_*t*−*τ*,*t*_−*f*_*t*−*τ*−1,*t*_; i.e., the forecast for month *t* made in month *t*−*τ* (*f*_*t*−*τ*,*t*_) equals the forecast for month *t* made in period *t*−*τ*−1 (*f*_*t*−*τ*−1,*t*_) plus the forecast update (*ϵ*_*t*−*τ*,*t*_). For a given forecast horizon of *H* months and a given long-term (i.e., beyond *H* months in the future) forecast ${\tilde{\mu }}_{t}$ (which depends on the month of the year), the iterative use of the above equation implies that the actual mean MUAC-Z in the absence of food assistance in month *t* equals ${\tilde{\mu }}_{t}+\sum_{\tau =0}^{H}\epsilon_{t-\tau ,t}$.

The forecast evolution model is defined by the probabilistic assumptions imposed on the vector (*ϵ*_*t*,*t*_,*ϵ*_*t*,*t* + 1_,…,*ϵ*_*t*,*t* + *H*_) of forecast updates in each month *t*, and the classical MMFE model [17] assumes that these forecast updates form an independent and identically distributed sequence of normal random vectors with mean zero. However, in an attempt to fit a model with this independence assumption, we found that our data exhibit significant correlations among forecast updates made in different months for the same target month. This lack of independence among forecast updates for the same target month means that the forecasting method—as with any real method—is not perfect (e.g., it could be due to serial correlation of weather deviations from seasonal means), and future forecast updates can be partially predicted from past forecast updates for the same target month. Hence, we generalize the MMFE model by incorporating two kinds of covariance among forecast updates: covariance among forecast updates for the same target month, given by $Cov(\epsilon_{t-\tau_{1},t},\epsilon_{t-\tau_{2},t})=\Sigma_{\tau_{1},\tau_{2}}^{1}$, and covariance among updates made in the same month, given by $Cov(\epsilon_{t,t+\tau_{1}},\epsilon_{t,t+\tau_{2}})=\Sigma_{\tau_{1},\tau_{2}}^{2}$. All other pairs of updates are assumed to be uncorrelated.

The first two years of data are used to estimate the parameters of the regression model and the remainder of the data are used to estimate the covariance matrices Σ^1^ and Σ^2^, where regularization is performed by applying factor analysis to guarantee stationarity of the forecast update process and shrinkage is used to compensate for the small sample size (§B.3 of S1 File).

### Problem Formulation

Our model follows a total of *N* = 2236 children equally distributed in age from 6-59 months for a duration of *T* = 120 months, where children who reach 60 months of age exit the system and are replaced by 6-month old children (infants under 6 months do not typically eat solid food and so are excluded). The decision variables in each month *t* are the number of kg of food assistance delivered by cash-based interventions and by transoceanic shipment, denoted by $O_{t}^{f}$ and $O_{t}^{s}$, respectively (f = fast, s = slow). These two modes of delivery have constant delivery lead times of *L*_*f*_ = 3 months and *L*_*s*_ = 6 months (they have been estimated to be 12 weeks and 23 weeks for Kenya in Fig 1 of [8]), respectively. Food assistance is divided evenly among all children and is consumed in the month that it is delivered.

In any month *t*, we assume that MUAC-Z values have a normal distribution with mean *μ*_*t*_ and known standard deviation *σ* = 0.62 (§B.2 of S1 File), where the latter is independent of the amount of food assistance (which is reasonable under our assumption of blanket distribution of food) and the month of the year, and

$$\begin{array}{c} \mu_{t}={\tilde{\mu }}_{t}+\sum_{\tau =0}^{H}\epsilon_{t-\tau ,t}+\frac{d}{N}\sum_{\tau =0}^{53}\frac{54-\tau }{54}\left(O_{t-\tau -L_{f}}^{f}+O_{t-\tau -L_{s}}^{s}\right). \end{array}$$ (1)

The first two terms on the right side of Eq (1) give the mean MUAC-Z in the absence of food assistance, as derived from the MMFE model. In the last term in Eq (1), the effect of food assistance decreases by 1/54^th^ of its original value every month because the 59-month old children (who comprise 1/54^th^ of the child population) exit the system and are replaced by 6-month old children.

A child with MUAC-Z value *z* is assumed to have a monthly mortality rate (i.e., the probability of dying within one month) of *e*^*a*−*bz*^, where *a* = −7.13 and *b* = 0.722, based on a meta-analysis (due to the intricate relationship between disease, undernutrition and mortality, this meta-analysis was restricted to longitudinal studies) of the relationship between monthly mortality and weight-for-age Z score (WAZ) in sub-Saharan Africa and Asia [18] (§3.1 of S1 File) and on the observation that the relationship between mortality and WAZ is very similar to the relationship between mortality and MUAC-Z (Table 2 of [21]). The shipments $O_{t}^{f}$ and $O_{t}^{s}$ are chosen to minimize the expected total number of child deaths over months 1,…,*T*.

Our decision variables $O_{t}^{f}$ and $O_{t}^{s}$ are each subject to a budget constraint that is impacted by U.S. food assistance policy regarding cash-based assistance and agricultural cargo preference. Let *c*_*f*_ and *c*_*s*_ be the cost/kg of food assistance, which includes commodity and transportation costs, for the two procurement modes. Based on the cost of cereals in Table 5 of [8], we set *c*_*f*_ = $0.363/kg for cash-based interventions and let *c_s_* $0.567/kg plus the shipping cost for transoceanic shipments. Compliance with the agricultural cargo preference in 2006 (which was then set at 75% US-flag carriers) was estimated to increase shipment costs by 46% [13], implying that U.S.-flag carriers were 61.3% more costly than competitive shipping (i.e., 0.75(1.613)+0.25 = 1.46). Hence, if the shipping cost of $0.228/kg in Table 5 of [8] represents compliance with using 75% US-flag carriers, then the competitive shipping cost rate is $0.228/1.46 = $0.156/kg. Denoting the allowable proportion of ships to be non-U.S. flag carriers by *p*, we can express the food assistance cost per kg for transoceanic shipments by *c_s_* = 0.567 + 0.156[*p* + (1−*p*)1.613] = $0.819−$0.096*p*. Weallow *p* to vary between 0.25 and 1.0, where 0.5 is the current value, 0.25 was the value between 1985 and 2012, and 1.0 corresponds to eliminating the cargo preference restriction.

Let *B* be the annual food assistance budget. We assume that *l* is the maximum allowable proportion of food that is delivered via cash-based interventions, so that the annual budgets for the fast and slow shipments modes are $\frac{c_{f}l}{c_{f}l+c_{s}(1-l)}B$ and $\frac{c_{s}(1-l)}{c_{f}l+c_{s}(1-l)}B$, respectively. We allow *l* to vary between 0.65 and 1.0, where 0.65 corresponds to the current portion of WFP’s shipments that are in the form of cash-based interventions (Fig 2 of [6]) and 1.0 corresponds to the U.S. shifting entirely from transoceanic shipments to cash-based interventions. Let $B_{t}^{f}$ and $B_{t}^{s}$ be the budgets for the fast and slow shipment modes for the remainder of the fiscal year as of month *t*. At the beginning of the fiscal year (i.e., for *t* = 1,13,25,…), we have $B_{t}^{f}=\frac{c_{f}l}{c_{f}l+c_{s}(1-l)}B$ and $B_{t}^{s}=\frac{c_{s}(1-l)}{c_{f}l+c_{s}(1-l)}B$, and the remaining budgets evolve throughout the fiscal year according to

$$B_{t+1}^{f}=B_{t}^{f}-c_{f}O_{t}^{f},$$ (2)

$$B_{t+1}^{s}=B_{t}^{s}-c_{s}O_{t}^{s}.$$ (3)

We assume that the remaining budgets are exhausted in the last month of each fiscal year.

### The Annual Budget

Our value for the annual budget is derived by assuming that the actual amount of food assistance consumed per year by the 2236 children in [16] was delivered under the pre-2012 values of *l* = 0.65 and *p* = 0.25. In the 42 communities under study, 266.8k kgs/year were delivered on average and 25.3% of the people were children. Assuming that the average child between six months and five years of age consumes half as many calories as an adult [22], we calculate that the 2236 children in [16] received 38,636 kg/yr; at 3679 kcal/kg, this is an average of 174.2 kcal/day per child, which is somewhat less than the typical supplementary feeding dose of 250 kcal/day [23]. The cost of this food is (0.363(0.65) + [0.819−0.096(0.25)]0.35)38,636 = $19,867/yr.

### Computational Approach

The resulting optimization problem is a stochastic dynamic program [24] with a 165-dimensional system state, consisting of the 108 previous orders ($O_{t-54}^{f},\ldots ,O_{t-1}^{f}$, $O_{t-54}^{s},\ldots ,O_{t-1}^{s}$), the 55 previous forecast updates (*ϵ*_*t*−*H*,*t*_;*ϵ*_*t*−*H*+1,*t*_,*ϵ*_*t*−*H*+1,*t*+1_; …;*ϵ*_*t*−1,*t*_,*ϵ*_*t*−1,*t*+1_,…,*ϵ*_*t*−1,*t*+*H*−1_; *ϵ*_*t*,*t*_,*ϵ*_*t*,*t*+1_,…,*ϵ*_*t*,*t*+*H*_), and the current available budgets ($B_{t}^{f},B_{t}^{s})$. Due to the so-called curse of dimensionality [24], we cannot compute the optimal solution. Instead, we find a closed-form solution to a simpler version of the problem that has only a single shipment mode and allows negative orders (§C.2 of S1 File). Motivated by the fact that this optimal solution is affine in the system state (Theorem 1 in the S1 File), we numerically compute the death-minimizing policy to the original problem among ordering policies that are affine (within a specific class that has 12 parameters to be optimized—see Eq (77) in S1 File) in the state variables. Other computational details appear in §C.3 of S1 File.

## Results

### Forecasting Model

As in [16], our focus is forecasting and we are not attempting any inference with respect to the parameters of the regression model; hence, we do not present the results of the regression model; indeed, the only information we need to know about the regression model for our optimization problem is contained in the parameters of the forecast evolution model. However, we do note that the results of the calculations described at the end of §1.2 of the S1 File estimate that the food assistance is associated with a reduction in the mean MUAC-Z in the population of 1.4, suggesting that food assistance is very important for the health of the children in this region.

### Forecast Evolution Model

In the estimate of Σ^1^ (Fig D in S1 File), which is the covariance matrix of forecast updates made for the same target period, the largest absolute values are along the diagonal, implying that covariances between pairs of updates made in different periods for the same target period are not strongly correlated with each other. In addition, the first updates, which correspond to a lead time of nine months, exhibit a small amount of positive correlation with all future updates, meaning that the first forecast is usually too conservative and future forecast updates tend to move the forecast in the same direction. Our analysis shows that the predicted accuracy of the forecasting method does not match the observed accuracy unless we incorporate the off-diagonal terms of Σ^1^. The largest matrix entries of Σ^1^ are at positions (0,0) and (9,9), which are the variances of the last and first updates, respectively. Hence, the first and last forecast updates are the most informative, and each of the other forecast updates provides a relatively modest amount of information. Nonetheless, these other forecast updates are still important because their impact on forecast error accumulates over time.

The primary characteristic of our estimate of Σ^2^ (Fig E in S1 File) is that forecast updates made in the same period for different future target periods tend to be slightly positively correlated with each other. This feature could be explained by the fact that the same factors (e.g. change in weather patterns) determine these updates.

In our context, the most important property of the forecasting method is its accuracy. We measure accuracy by the root mean square error (RMSE) and compare the observed forecast accuracy for different forecast lead times with the forecast accuracy predicted by our model (Eq (13) in S1 File). The close fit of the true RMSE, the model RMSE and the RMSE of the model estimated without regularization (Fig F in S1 File) suggests that our model describes the accuracy of the forecasting method well, and regularization has only a small impact on forecast accuracy.

### Main Results

**Fig 2:**
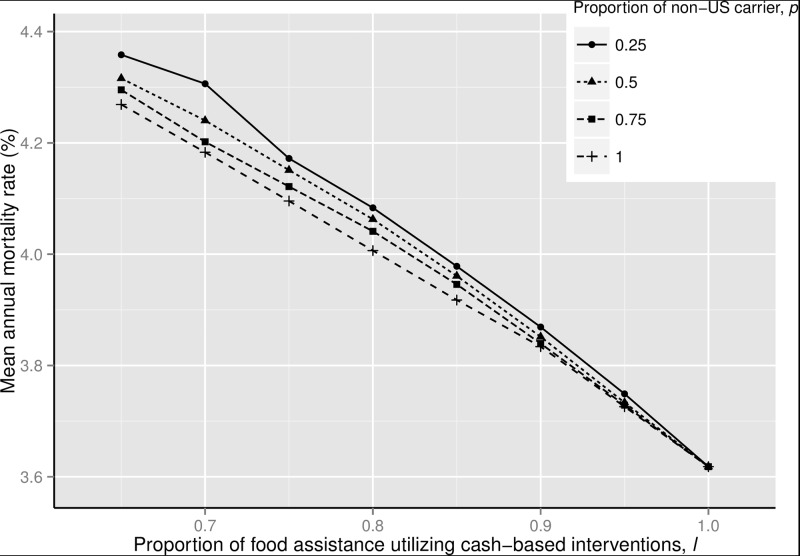
Dependence of the annual mortality rate on the proportion of food assistance utilizing cash-based interventions (*l*) and the proportion of transoceanic shipments employing non-US-flag carriers (*p*). The current U.S. policy is represented by *l* = 0.65 and *p* = 0.5, the elimination of the U.S.-flag vessel requirement corresponds to *p* = 1.0, and *l* = 1.0 corresponds to the U.S. switching entirely to cash-based interventions.

We compute the annual child mortality rate in the population of 2236 children as a function of the food assistance delivery parameters *l* and *p* (Fig 2). Because the cost of transoceanic shipments only influences the non-cash portion of food assistance, the curves in Fig 2 fan out to the upper left from a single point in the lower right. The annual mortality rate decreases from 4.4% to 3.7% (a 16.2% relative reduction) as we switch from the current U.S. policy (*l* = 0.65 for cash-based interventions, *p* = 0.5 for non-U.S. carriers) to a pure cash-based policy (*l* = 1). Nearly all of the improvements in Fig 1 are due to increasing the use of cash-based interventions: at *l* = 0.65, where the cargo preference parameter *p* has its maximum impact, increasing *p* from 0.25 to 1 decreases the annual mortality rate from 4.5% to 4.4% (a 2.0% relative reduction).

Our model is based on the estimated parameters, which are subject to statistical uncertainty. We quantify the impact of uncertainty on our main results by performing a Monte Carlo simulation with 1000 repetitions. We focus on uncertainty in parameters which are estimated in this paper: the two covariance matrices, the month-specific means of MUAC-Z and the variance of MUAC-Z in the population. For each run of the Monte Carlo simulation, we draw new values of the estimated parameters from the corresponding sampling distributions of the estimators, which are normal distributions for the MUAC-Z means and Wishart distributions for the two covariance matrices and the variance of MUAC-Z. The sampling distributions are assumed to be independent of each other. For each set of drawn parameter values, we simulate the performance of the procurement policy optimized for the nominal set of parameter values. This allows us to simulate the uncertainty in the actual performance of the policy that was derived based on nominal parameter values. The 95% confidence intervals are computed as the 2.5^th^ and 97.5^th^ percentiles of the empirical distributions of simulated mean annual mortality rates.

For the *p* = 0.5 and *p* = 1.0 curves in Fig B, Figs G(a-b) in S1 File show the 95% confidence intervals for the mean annual mortality rate, and Figs G(c-d) in S1 File give the 95% confidence intervals for the absolute reduction in the mean annual mortality rate relative to the *l* = 0.65 case. The confidence intervals are much wider in Figs G(a-b) in S1 File than in Figs G(c-d) in S1 File because sampling variability shifts the entire annual mortality rate curve up or down. Hence, conditional on a particular realization of estimated parameters, Figs G(c-d) in S1 File imply that an increase in *l* leads (with high confidence) to a decrease in the mean annual mortality rate.

Cash-based interventions are both cheaper and faster than transoceanic shipments, and so improved efficiency could be due to larger quantities procured because of the reduced cost of cash-based interventions, or to more efficient ordering based on more accurate forecasts because of shorter delivery lead times under the cash-based interventions. To isolate the impacts of cost and delivery lead time, we recompute the curves in Fig 2 under two sets of assumptions (fixing *p* = 0.5 in both cases): first, that the delivery lead times of transoceanic shipments and cash-based interventions are both three months (Fig H in S1 File), so that the only difference between the two procurement channels is the lower cost under cash-based interventions, and then under the assumption that the cost of cash-based interventions is the same as the cost of transoceanic shipments (we set *c*_*s*_ = *c*_*f*_ = (0.65(0.363) + 0.35[0.819−0.096(0.5)] = $0.506/kg) (Fig H in S1 File), so that the only difference between the shipment modes is the shorter lead time of cash-based interventions. These results imply that the main driver of the mortality reduction associated with cash-based interventions is reduced cost, not reduced delivery lead time.

### Sensitivity Analysis

We perform three sensitivity analyses, while fixing the cargo preference parameter *p* = 0.5 in the first two analyses. In the first analysis, we change the delivery lead times from the Kenya-specific values of *L*_*f*_ = 3 months and *L*_*s*_ = 6 months to *L*_*f*_ = 2 months and *L*_*s*_ = 5 months, which represent the respective averages of 8.5 weeks and 22.3 weeks over the nine countries considered in [8]. This modest reduction in delivery lead times has virtually no effect on the results (Fig I in S1 File). In the second analysis, due to the imprecision of the effect of food assistance, we re-analyze the problem with *d* = 0.0075/kg, which is half the effect of our base-case value of *d* = 0.015/kg. The annual mortality rates are higher in this case (Fig J in S1 File), and the relative reductions in the annual mortality rates are approximately half of what they are in Fig 2.

The third sensitivity analysis considers an alternative estimate of the annual food budget, which uses the worldwide supply-to-demand ratio of food assistance instead of the actual amount of food delivered in [16]. If we assume that 790.7M people (adults and children) in developing countries are undernourished [25], the cost to feed one person for one year is $40 [26], and the WFP spends $3.8B on food assistance (as in 2012 [3]), then the supply-to-demand ratio of food assistance is $\frac{3.8\text{B}}{790.7\text{M}(40)}=0.120$ (i.e., the WFP is supplying only 12.0% of the food necessary to feed all undernourished people, which is not inconsistent with an estimate that 16% of consolidated appeals for emergencies in sub-Saharan Africa are funded [2]). Assuming that the percentage of undernourished children is the same as the percentage of children with Z values below -2 (Fig 9 of [27]), and using the counterfactual estimate that 70.8% of children in the 42 communities in [16] would have MUAC-Z <−2 in the absence of food assistance (note that 22.8% of the children in [16] had MUAC-Z values below -2, which is similar to the 23.8% of people in Sub-Saharan Africa who are undernourished [25]), we assume that the annual budget is enough to feed 0.12(0.708)(2236) = 190 children. Assuming a dose of 250 kcal/day [23] per child and 3679 kcal/kg, we feed each child 24.8 kg/yr, for a total of 4712 kg/yr. At post-2012 values of *l* = 0.65 and *p* = 0.5, the cost of this food is (0.363(0.65) + [0.819−0.096(0.5)]0.35)4712 = $2382/yr. In summary, this more restrictive budget is much smaller than the base-case budget of $19,867/yr, primarily due to the assumption that only 12% of needy—rather than all— children require food, but also because this budget has a smaller dose (174.2 vs .250 kcal/day) and a slightly smaller cost (*p* = 0.25 vs. 0.5).

The results under this more restrictive budget (Fig K in S1 File) are qualitatively identical to those under the base-case budget in Fig 2, except that the mortality reductions are much smaller: switching from the current U.S. policy (*l* = 0.65, *p* = 0.5) to *l* = 1 achieves a relative reduction in the annual mortality rate of only 2.2%, compared to 16.2% under the base-case budget. Even though each kg of food has a higher impact under the restrictive budget (a reduction in the annual mortality rate of 1.39% per kg/person-mo under the restrictive budget and 0.60% per kg/person-mo under the base-case budget), the restrictive budget provides far less food than the base-case budget (0.06 kg/person-mo vs. 0.61 kg/person-mo), which impedes the leverage of the cash-based interventions.

## Discussion

### Results

As noted earlier, the international food assistance landscape has been changing quickly, and for the better. But there remains much room for improvement in relaxing the direct transfer in-kind and cargo preference requirements still built into Title II emergency food aid, which remains the workhorse program for U.S. food aid. Our study directly tackles this issue and generates two main results. First, we estimate that child mortality in northern Kenya can be reduced by 16.2% (i.e., from 4.4% to 3.7%) if the U.S. joined the rest of the world and switched entirely to cash-based interventions such as LRP, cash transfers and food vouchers; to our knowledge, there are no other analyses that directly assess the impact of these changes on child mortality. Although our forecasting data are from Kenya, the results are likely to be representative of sub-Saharan Africa (recall that 22.8% of the children in [16] had MUAC-Z values below -2, which is similar to the 23.8% of people in Sub-Saharan Africa who are undernourished [25]). The relative child mortality reduction achieved by a change in U.S. policy is approximately linear in the food budget, as can be seen by comparing the results under the base-case budget and the restrictive budget considered in the sensitivity analysis: the budget ratio is 2382/19,867 = 0.120 and the relative mortality reduction ratio is 2.2/16.2 = 0.136. To the extent that spatial targeting is efficient (i.e., areas with significant mortality receive significant amounts of food assistance), the 16.2% estimate should be indicative of the overall relative reduction in child mortality in sub-Saharan Africa. Given that the United Nations Millenium Development Goal 4, which is to reduce child mortality by 67% between 2000-2015, was not achieved in most countries [28], a change in U.S. policy might help accelerate progress on this crucial metric.

The second main result is that the effect of relaxing the cargo preference restriction is dwarfed by the effect of increasing cash-based interventions. More specifically, eliminating the cargo preference restriction (i.e., using all non-U.S.-flag vessels for transoceanic shipments) would achieve the same child mortality reduction as increasing cash-based intervention use from 0.65 to 0.69. Note that the cargo preference restrictions and the cash-based intervention restrictions are not two alternative policies, but rather are closely connected: as can be seen in Fig 2, the move to cash-based assistance renders cargo preference restrictions largely irrelevant because much of the food would be purchased in developing countries, obviating the ocean freight problem. Hence, U.S. policymakers interested primarily in minimizing child mortality should focus their efforts on relaxing the restrictions on cash-based interventions.

Our results also highlight two other issues. The MMFE parameter estimates (Fig D in S1 File) suggest that the power of the forecasting tool from [16] comes from the updates in the first and last (ninth) months, which may be useful when considering other uses of this forecasting tool. In addition, for slow-onset emergencies, we find that the benefits from switching to cash-based interventions stem from cost reduction, not delivery acceleration (Fig H in S1 File). This finding suggests that prepositioning of food aid, which is an alternative approach to improve timeliness [29] but—in contrast to LRP—increases the cost of transoceanic food shipments by 25-40%, is not a cost-effective approach to slow-onset emergencies. Nonetheless, prepositioning may be appropriate for unforeseen disasters (e.g., tsunamis, earthquakes) [30].

### Limitations

The model and related data have some shortcomings that make it difficult to quantify the accuracy of our results. We estimate the increase in MUAC-Z from supplementary food by using randomized controlled trial data on food’s impact on WHZ. Similarly, we estimate the relationship between MUAC-Z and mortality using data on the relationship between WAZ and mortality, although the two relationships are very similar (Table 2 in [21]). The data in [16] uses MUAC-Z standards from the 1985 CDC/WHO growth charts rather than the more recent WHO charts [31]. We assume blanket distribution of food, which is the primary mode of food distribution in slow-onset disasters; analyzing a food allocation policy that targeted children with low MUAC-Z would be much more difficult because we would have to better understand the left tail of the MUAC-Z distribution.

Fitting the MMFE model in a nonstationary environment is problematic. Kenya experienced an exceptional drought in the year 2000, and we removed the first 12 months from the data set to provide a reasonable fit to the forecasting model in [16]. Even with this omission, the standard MMFE model did not provide a good fit to the forecasts, and we needed to incorporate off-diagonal terms of Σ^1^ because the initial forecast (for nine months in the future) was typically conservative, and subsequent forecast updates were positively correlated. In addition, the forecasting model in [16] has not been applied outside of the arid region of northern Kenya. It is difficult to assess whether—and how—the model’s forecasting performance would change if used in other parts of sub-Saharan Africa. However, Africa receives >80% of U.S. emergency food assistance and the primary recipients are countries with large arid and semi-arid regions (Chad, Ethiopia, Kenya, Mali, Niger, Somalia, South Sudan, Sudan) [12]. So our study region of northern Kenya is not atypical.

Finally, we assume that the cost and timeliness of LRP extend to cash transfers and food vouchers. While this assumption is likely to be conservative (cash and vouchers may be faster and less expensive than LRP), the optimal mix of cash, food or vouchers to deliver food assistance is a multi-faceted problem that includes the impact on local food prices, consumers and producers in urban and rural areas [7], and is beyond the scope of the present study. A range of studies have questioned whether in-kind food aid deliveries disrupt recipient food markets, to the detriment of local producers, or even help prolong civil conflict [2, 32–34]. These hypothesized effects help explain the mixed support of recipient countries for traditional, in-kind food aid. There is a literature documenting other impacts of this policy switch: e.g., consumers prefer cash-based assistance [35] and humanitarian agency personnel in conflict zones are at a reduced risk [36, 37]. These arguments helped motivate passage of the Global Food Security Act of 2016 (S. 1252) by the U.S. Congress in July 2016 that permanently authorizes the Emergency Food Security Program by which the U.S. now provides cash-based food assistance along the lines we recommend.

Taken together, despite our considerable effort to capture the forecasting, lead time and ordering interactions, the main driver of our results is the different cost of the two procurement modes (i.e., *c*_*f*_ and *c*_*s*_ in Table 1). These estimates are reasonably accurate, and suggest that our second main result—that the impact of restrictions on cash-based interventions is far greater than the impact of cargo preference restrictions—is quite robust. However, the first main result—that the elimination of the restriction on cash-based interventions would decrease child mortality by 16.2%—is less precise.

## Conclusion

We estimate that the restriction on the use of cash-based interventions (LRP, cash transfers and food vouchers) is much more consequential than the agricultural cargo preference requirement, and that a shift by the U.S. to rely entirely on cash-based interventions for its food assistance distribution could reduce child mortality by 16.2%. This improvement is due primarily to the reduced cost of cash-based interventions, not to the reduced delay.

## Supporting Information

S1 File Supporting Material. Contains additional figures and tables discussed above.
(PDF)

S2 File Raw Data. Contains the data used to perform this analysis.
(DTA)
