# Supplementary material for: Assessing the Impact of U.S. Food Assistance Delivery Policies on Child Mortality in Northern Kenya
Source: PLoS One. 2016 Dec 20;11(12):e0168432. doi: 10.1371/journal.pone.0168432 (PMC5173367; doi:10.1371/journal.pone.0168432)
Supplement: S1 File — Contains additional figures and tables discussed above. (PDF) [file pone.0168432.s001.pdf]

Supporting Material

We describe the relevant data and the forecasting model in §A and the forecast evolution model in §B. We optimize the order quantity in §C. Figs A-K are referred to in the main text.

A Forecasting Methodology

We use the data and methodology described in [1] to forecast childhood undernutrition in northern Kenya. The data are briefly described in §A.1, the effect of food assistance is calculated in §A.2, and the methodology is given in §A.3.

A.1 Data

The data, which are described in detail in [1], include community-level distributions of mid-upper arm circumference (MUAC) from children ages 6 to 59 months, which is a measure of child undernutrition. All MUAC values were transformed into standardized Z-scores referred to as MUAC-Z scores, using the 1978 CDC/WHO growth charts. Other data were aggregated at the community level, and include detailed livestock information (herd sizes, mortality rates, lactation rates and managed off-take rates for large and small livestock), food aid (the fraction of households receiving Unimix and regular cereals, as well the amount received per household), and biophysical variables (rain, forage availability, and the normalized differenced vegetation index, which is a numerical measure of the relative density of green vegetation obtained from satellite data).

The above data were collected monthly from February 2000 to May 2005 in 56 communities in northern Kenya. However, we discarded data from some time periods and some communities. In 2000 northern Kenya experienced an exceptional drought, causing the dynamics of MUAC-Z in the year 2000 to differ substantially from other years. Consequently, we removed the first 12 months from the data set to ensure that our forecasting method

works better in unexceptional years, which are far more common; hence, we use only 52 months of data. Furthermore, some observations were missing in each month, and imputing them decreases the quality of predictions, as measured by the root mean square error (RMSE) compared to the forecasts based on an unbalanced panel with missing observations. We removed 14 communities from our data set because of high levels of missing data (our threshold was 25% of observations in a period). The community-level panel is very unbalanced (Table 1 in [1]) due to poor data collection, entry and storage protocols of the Arid Land's Resource Management Project team of the Government of Kenya. Administrative problems do not appear to have been related to characteristics of the communities, but rather were due to internal management issues. The 2236 children in the remaining 42 communities (Table A) represent 76% of the total population of children in the 56 communities, and we hereafter restrict our analysis to this subset of communities.

A.2 The Effect of Food Assistance

As noted in the main text, because we will be using the MUAC-Z forecasts to optimize the food deliveries, we modify our training sample of MUAC-Z values so that the forecasts are calibrated to forecast the mean MUAC-Z net of the effect of the food assistance that was actually provided. To get this net MUAC-Z value, for each observation we find the amount of food assistance delivered to the community in each of the last 54 months and then subtract the effect of each portion of this food assistance from the observed realization of MUAC-Z. To perform these calculations, we need to address two issues: missing data and the arrival and departures of children in our sample.

If we do not observe the food assistance delivered in some month within our sample dates, we infer it using a linear model with dummy variables for the month and community (we tried a multiplicative model instead of a linear model, but the linear model produced a better fit). The amount of food assistance that was delivered to a community in a month before the earliest date of our sample is approximated by the mean observed amount of food

assistance delivered to that community during the dates covered by our sample.

Turning to the second issue, even though we assume that the effect of food assistance is persistent, 60-month old children age out of our sample and are replaced by 6-month old children, who have not yet received any food aid. These child replacements cause the effect of food assistance in a given month on the mean MUAC-Z of the children in our sample to gradually decrease over the following months. In particular, every month this effect decreases by $1/54^{\text{th}}$ of its original value because children of ages $5 - 59$ months are included in the sample and every month the 59-month-old children (who constitute $1/54^{\text{th}}$ of the sample because we assume that children are born uniformly over time) age out of our sample and are replaced by 6-month-olds. Therefore, the effect of food assistance on the sample mean τ periods after it was distributed is $\frac{54-\tau}{54}$ of the immediate effect of food assistance.

Finally, our data set provides the amount of food assistance in kilograms, but the food trials (e.g., [3]) measure food in calories. We convert kilograms into kcals by using the data from [2], which states that in 2012, one ton of average food assistance in Kenya satisfied annual caloric requirements for 4.8 people. The daily caloric requirement is 2100 kcal per person, and so 1 kg of food assistance contains on average $4.8(2100)(365)/1000 = 3679$ kcal. As consistency checks, we note that wheat contains 3400 kcal/kg, and Unimix contains 3800 kcal/kg. Recalling from the main text that 500 kcal/day over 90 days increases MUAC-Z by 0.19 [3], we find that 1 kg of food aid increases MUAC-Z by $\frac{3679}{500}\frac{0.19}{90} = 0.015$ on average, which we denote by d.

A.3 Regression Model

Our dependent variable is $Y_{i,t+\tau}$, which is the mean MUAC-Z in the absence of food assistance in community $i = 1, \ldots, 42$ in month $t + \tau$, as estimated in month $t = 1, \ldots, 52$. For a fixed i and t, we have 66 explanatory variables denoted by X_{jit} for $j = 1, \ldots, 66$, which includes lagged values of the MUAC moments, herd variables, food aid and biophysical variables; see Table 2 for a description of these variables, which is taken from Table 2 in [1]. For a fixed

forecast lead time τ, the regression equation is given by (our notation differs slightly from the equation on page 331 of [1])

$$Y_{i,t+\tau} = \beta_0 + \sum_{j=1}^{66} \beta_j X_{jit} + u_i + \phi_{(t+\tau)\text{mod}12} + v_{it}, \qquad (1)$$

where β_0 is a constant, u_i is the community-specific effect for $i = 1, \ldots, 42$, $(\phi_0, \ldots, \phi_{11})$ captures seasonality related to the month of the year, and v_{it} is the unobserved error term, which has mean zero and variance σ^2. We perform a different regression for each value of the forecast lead time τ, but we suppress this dependence on τ in the parameters $(\beta_0, \beta_{jit}, u_i, \phi_t, \sigma^2)$ in (1) for ease of presentation.

Because there are 119 explanatory variables in our regression, we regularize the estimation procedure by including a ridge penalty [4] with a coefficient chosen by five-fold cross-validation (i.e., the original sample is randomly split into five equal-sized parts, and we repeat the following procedure five times: use four parts as the training data to estimate the forecasting model and use the fifth part as the test data to make forecasts and compute the RMSE). The resulting RMSE was indistinguishable from the RMSE of the model without regularization.

As explained in [1], the estimation procedure might suffer from an endogeneity problem due to correlation between food assistance shipments and unobserved factors. But our main objective is to obtain the best possible predictions (in the RMSE sense), and we sacrifice the causal interpretation of regression coefficients to achieve this; consequently, we will not be interpreting the estimated coefficients.

The forecast accuracy is evaluated by comparing the forecast to an actual realization of mean MUAC-Z values in the absence of food assistance. Five time lags of biophysical variables are included among the predictors, which requires five consecutive observations in the same community to make a forecast for this community. Because the missing data are serially correlated, this requirement does not drastically reduce the amount of available

data: $\approx 50\%$ of the observations in our data set can be retro-forecasted to assess the forecast accuracy.

In a real forecasting setting, in each time period forecasts are typically made based on all the data available at that time. To mimic this state of affairs, we re-estimate our forecasting model each month using the data observed up to that time.

Applying our forecasting methodology to the forecast lead times $\tau = 1, \ldots, 12$ (Fig A) shows that the RMSE increases until nine months, suggesting that most of the useful information is revealed at most nine months in advance of the forecast target date. Consequently, we set the forecast horizon equal to nine months in §B.

While our approach generates forecasts at the community level, our ultimate goal is the optimization of food assistance shipments into the whole northern Kenya region. Consequently, in each month we aggregate the forecasts for individual communities by using a weighted average of the community forecasts, where the weight for community i, w_i, is the proportion of the population in the 42 communities that is from community i (Table 1). That is, if $f^{(i)}_{t,t+\tau}$ is the forecast for community i made in month t for month $t+\tau$, the aggregated forecast for the entire northern Kenya region is given by

$$f_{t,t+\tau} = \sum_{i=1}^{42} w_i f^{(i)}_{t,t+\tau}. \tag{2}$$

If we denote the standard deviation of the forecast $f^{(i)}_{t,t+\tau}$ by $\sigma^{(i)}_{t,t+\tau}$, then the variance of the aggregated forecast in (2) is given by

$$\begin{aligned} \sigma^2_{t,t+\tau} &= \frac{\sum_{i=1}^{42} w_i \sigma^{(i)2}_{t,t+\tau}}{\sum_{i=1}^{42} w_i} + \frac{\sum_{i=1}^{42} w_i (f^{(i)2}_{t,t+\tau} - f^2_{t,t+\tau})^2}{\sum_{i=1}^{42} w_i}, & (3) \\ &= \frac{\sum_{i=1}^{42} w_i (f^{(i)2}_{t,t+\tau} + \sigma^{(i)2}_{t,t+\tau})}{\sum_{i=1}^{42} w_i} - f^2_{t,t+\tau}. & (4) \end{aligned}$$

Unless otherwise specified, only forecasts aggregated across communities will be discussed.

B Forecast Evolution Model

The forecast evolution model is formulated in §B.1, the long-term mean MUAC-Z in the absence of food assistance and the standard deviation of the population-wide MUAC-Z in the absence of food assistance are estimated in §B.2, and the covariance matrices of the forecast evolution model are estimated in §B.3.

B.1 The Model

Recall that $f_{t-\tau,t}$ is the forecast made in month $t-\tau$ of the mean MUAC-Z in month t in the absence of food assistance. The evolution of the forecasting model in §A.3 can be described in terms of the forecast updates,

$$\epsilon_{t-\tau,t} = f_{t-\tau,t} - f_{t-\tau-1,t}, \tag{5}$$

which is the amount that the forecast for month t is adjusted (either upward or downward) as we move from month $t-\tau-1$ to month $t-\tau$. Put another way, the forecast for month t made in month $t-\tau$ (i.e., $f_{t-\tau,t}$) equals the forecast for month t made in month $t-\tau-1$ (i.e., $f_{t-\tau-1,t}$) plus the forecast update (i.e., $\epsilon_{t-\tau,t}$). We are only considering forecast updates made up to nine periods ahead (Fig A), and assume that any forecast for month t made more than nine months ahead is $\tilde{\mu}_{t\text{mod}12}$, which is the long-term mean MUAC-Z in the absence of food assistance in the month corresponding to time period t. Hence, the first forecast for the mean MUAC-Z in month t using the regression model is made in month $t-H$ and is $f_{t-H,t} = \tilde{\mu}_{t\text{mod}12} + \epsilon_{t-H,t}$. In the following month, we change the forecast to $f_{t-H+1,t} = f_{t-H,t} + \epsilon_{t-H+1,t} = \tilde{\mu}_{t\text{mod}12} + \epsilon_{t-H,t} + \epsilon_{t-H+1,t}$, and so on. Using equation (5) in an iterative manner, we find that the realized mean MUAC-Z in month t in the absence of

food assistance, denoted by y_t (which also equals $f_{t,t}$), can be expressed as

$$y_t = \epsilon_{t,t} + f_{t-1,t}, \tag{6}$$

$$= \epsilon_{t,t} + \epsilon_{t-1,t} + f_{t-2,t} = \dots, \tag{7}$$

$$= \sum_{\tau=0}^{H} \epsilon_{t-\tau,t} + \tilde{\mu}_{t\text{mod}12}. \tag{8}$$

Thus far, the forecast evolution model in (5)-(8) is identical to the Martingale Model of Forecast Evolution (MMFE) [5], aside from the fact that we incorporate seasonality into the long-term forecast, $\tilde{\mu}_{t\text{mod}12}$. The MMFE model assumes that the vector $(\epsilon_{t,t}, \epsilon_{t,t+1}, \dots, \epsilon_{t,t+H})$ of forecast updates made in month t form an independent and identically distributed (iid) sequence of normal random vectors. As noted in the main text, we generalize the MMFE model by incorporating two kinds of covariance among forecast updates: covariance among forecast updates for the same target month, given by $Cov(\epsilon_{t-\tau_1,t}, \epsilon_{t-\tau_2,t}) = \Sigma^1_{\tau_1,\tau_2}$, and covariance among updates made in the same month, given by $Cov(\epsilon_{t,t+\tau_1}, \epsilon_{t,t+\tau_2}) = \Sigma^2_{\tau_1,\tau_2}$. All other pairs of updates are assumed to be uncorrelated: $Cov(\epsilon_{t_1,t_2}, \epsilon_{t_3,t_4}) = 0$ unless $t_1 = t_2$ or $t_3 = t_4$. In our model, forecast updates are assumed to have zero mean and have a multivariate normal distribution with the covariance structure described above. Note that the diagonals of the 10×10 covariance matrices Σ^1 and Σ^2 are the same by construction. Also, in the classical MMFE model, the matrix Σ^1 is diagonal – and hence the covariance is characterized by Σ^2 – because there are no correlations among forecast updates made in different months.

The mean squared error of a forecast with lead time k is

$$E[(y_t - f_{t-k,t})^2] = E\left[\left(\tilde{\mu}_{\text{tmod12}} + \sum_{\tau=0}^{H+1} \epsilon_{t-\tau,t} - \tilde{\mu}_{\text{tmod12}} - \sum_{\tau=k}^{H+1} \epsilon_{t-\tau,t}\right)^2\right], \tag{9}$$

$$= E\left[\left(\sum_{\tau=0}^{k-1} \epsilon_{t-\tau,t}\right)^2\right], \tag{10}$$

$$= Var(\sum_{\tau=0}^{k-1} \epsilon_{t-\tau,t}), \tag{11}$$

$$= \sum_{\tau_1=0}^{k-1} \sum_{\tau_2=0}^{k-1} Cov(\epsilon_{t-\tau_1,t}, \epsilon_{t-\tau_2,t}), \tag{12}$$

$$= \sum_{\tau_1=0}^{k-1} \sum_{\tau_2=0}^{k-1} \Sigma^1_{\tau_1,\tau_2}. \tag{13}$$

B.2 Estimating the Long-term Mean and the Standard Deviation

We estimate the long-term MUAC-Z mean for each month of the year in the absence of food assistance ($\tilde{\mu}_{\text{tmod12}}$) by taking a population-weighted average of MUAC-Z means in each month and averaging the result across years. The mean MUAC-Z ranges from a low of -2.43 in October to a high of -2.28 in February (Fig B). we observe that changes in the mean of MUAC-Z are significantly larger than changes in its standard deviation. The standard deviation of MUAC-Z is estimated by taking a sample analogue of (4). Because these standard deviations have very little variation across months, we assume that the standard deviation is independent of the month of the year. Averaging across months yields $\sigma = 0.62$.

B.3 Estimating the Covariance Matrices

We use the first two years of data to estimate the parameters in the regression model in (1), and then use the remainder of the data to generate forecasts up to nine months ahead. Forecasts are generated for individual communities and then aggregated into forecasts for the entire region using (2). Sequential forecasts are turned into forecast updates by taking

first differences according to equation (5), and the covariance matrices Σ^1, Σ^2 are estimated from these forecast updates.

The sample covariances used to estimate Σ^1 and Σ^2 are regularized to ensure stationarity of the forecast update process and to account for the small sample size (we have only 33 observations to estimate 55 covariance parameters in each matrix). More specifically, regularization is performed by sequentially applying factor analysis and shrinkage to the sample covariance matrices Σ^1 and Σ^2, where factor analysis is used to guarantee stationarity of the forecast updates process and shrinkage is used to compensate for the small sample size.

Factor analysis attempts to address the possibility that the variation of observed forecast updates is driven by a small number of underlying unobserved random variables called factors. This method [6] approximates a covariance matrix Σ by the sum of a diagonal matrix Ψ and a low-rank matrix LL^T, where $L \in \mathcal{R}^{p \times k}$, $p = 10$ is the number of rows or columns in Σ, and $k < p$ is the rank of LL^T; the value of k is specified later in this subsection. Factor analysis regularizes the estimate by reducing the number of parameters to be estimated from $\frac{p(p+1)}{2}$ to $p + pk$.

Shrinkage works by estimating Σ by a weighted average of the sample covariance $\hat{S}$, which is a no-bias, high-variance estimator, and the identity matrix I, which represents a high-bias, minimum-variance estimator,

$$\hat{\Sigma} = (1 - \rho)\hat{S} + \rho \frac{Tr(\hat{S})}{p} I, \tag{14}$$

where $\mathrm{Tr}(\hat{S})$ is the trace of the matrix $\hat{S}$, and ρ is the shrinkage coefficient, which can range from 0 to 1. We use the Oracle-Approximating Shrinkage (OAS) estimator [7], which minimizes the expected matrix norm of an error within the class of shrinkage estimators (which includes the sample covariance as a trivial case with zero shrinkage), yielding the

optimal shrinkage coefficient

$$\rho = \frac{(1-\frac{2}{p})Tr(\Sigma \cdot \Sigma) + Tr^2(\Sigma)}{(n+1-\frac{2}{p})Tr(\Sigma \cdot \Sigma) + (1-\frac{n}{p})Tr^2(\Sigma)}, \tag{15}$$

where n is the sample size. As expected for a small-sample correction, $\rho \to 0$ in (15) and $\hat{\Sigma} \to \hat{S}$ in (14) as $n \to \infty$.

We cannot directly apply (15) because we do not know the true covariance matrix Σ. Instead, we use an iterative procedure, which switches between the estimation of Σ and the optimal ρ for 20 iterations (during which convergence was achieved), as follows:

1. Set $i = 0$ and initialize the estimate of the covariance matrix with the sample covariance matrix: $\hat{\Sigma}_0 = \hat{S}$.

2. Estimate of optimal shrinkage coefficient using the current estimate of the covariance matrix: $\rho_{i+1} = \frac{(1-\frac{2}{p})Tr(\hat{\Sigma_i}^2)+Tr^2(\hat{\Sigma}_i)}{(n+1-\frac{2}{p})Tr(\hat{\Sigma_i}^2)+(1-\frac{n}{p})Tr^2(\hat{\Sigma}_i)}$.

3. Update the covariance matrix estimate using the current estimate of the optimal shrinkage coefficient: $\hat{\Sigma}_{i+1} = (1-\rho_{i+1})\hat{S} + \rho_{i+1}\frac{Tr(\hat{S})}{p}I$.

4. If $i < 20$, go to step 2; otherwise, increment i by one and continue to step 5.

5. Stop and return $\hat{\Sigma}_{21}$ as the covariance matrix estimate, ρ_{21} as the optimal shrinkage coefficient.

To specify the rank k of the matrix LL^T in the factor analysis, we find for each k the minimum value of the shrinkage coefficient ρ that makes the forecast update process stationary. Fig C shows the boundary in the two-dimensional (ρ, k) space between forecast update stationarity and nonstationarity. We set $k = 3$, which reduces the number of parameters to be estimated from 55 to 40, while decreasing the minimum necessary shrinkage from 0.295 (the value in the absence of factor analysis) to 0.231. This reduction in ρ is desirable because too much shrinkage (i.e., a high value of ρ) can bias the off-diagonal elements of the estimate

towards zero. We set the shrinkage coefficient to $\rho = 0.232$, which is below the value of 0.246 derived in (15) because part of the regularization is performed through factor analysis, and obtain a stationary forecast update process with a maximum absolute eigenvalue equal to 0.98.

In summary, we sequentially apply factor analysis and shrinkage with rank $k = 3$ and shrinkage coefficient $\rho = 0.232$ to both Σ^1 and Σ^2, which generates sufficient regularization to ensure stationarity of the forecast updates process without strongly biasing the off-diagonal elements towards zero.

We conclude this subsection by describing how we deal with missing data. Nearly 50% of the forecasts for individual communities cannot be generated because the required data are missing. It is necessary to infer the missing forecasts to obtain the aggregated forecasts in (2). If a forecast made in month t for month $t + \tau$ and community i is missing, we substitute it with an average of the two forecasts for community i that are:

1. Made for the target month $t + \tau$ in the month closest to t, i.e. $f^i_{t+s,t+\tau}$, where $s \in \mathcal{Z}$ has the smallest absolute value for which observation $f^i_{t+s,t+\tau}$ is not missing (if there is a tie for the closest absolute value, an average of the two forecasts is used);

2. Made in month t for the target month $t + \tau + s$ closest to $t + \tau$, i.e. $f^i_{t,t+\tau+s}$, where $s \in \mathcal{Z}$ has the smallest absolute value for which observation $f^i_{t,t+\tau+s}$ is not missing (if there is a tie for the closest absolute value, an average of the two forecasts is used).

This simple inference method implicitly assumes that forecasts are smooth functions of the months when they are made and targeted for, and that data are missing at random.

We also tested two other approaches to infer the missing data: using the forecast from item 1 above (i.e., using only forecasts made in the same month) and using the forecast from item 2 above (i.e., using only forecasts for the same target month). All three inference methods lead to virtually the same covariance matrix estimates, highlighting the robustness of our approach.

C Optimizing the Food Assistance Decisions

In this section, we choose the monthly food assistance quantities to minimize child mortality subject to an annual budget constraint. Child mortality is specified in terms of MUAC-Z in §C.1. In §C.2, we derive a closed-form solution for the simplified problem that has a single delivery mode and allows monthly orders to be negative. This closed-form solution yields monthly quantities that are affine in all previous orders, all previously observed forecast updates, and the available budget. In §C.3, we return to our original problem, which has two delivery modes and requires monthly orders to be nonnegative. Motivated by the results in §C.2 and the fact that this problem is difficult to solve exactly, we numerically optimize over the restrictive class of policies that are affine in all previous orders, all previously observed forecast updates, and the current budget.

C.1 The Relationship Between MUAC-Z and Child Mortality

The goal of the optimization problem is to minimize the expected number of child deaths. We assume that the monthly mortality rate for a child with MUAC-Z value z is e^{a-bz} for parameters a and b. In [8], the monthly mortality rate is estimated to depend on weight-for-age Z-score (WAZ) via $e^{-7.13-0.722z}$, and we extrapolate this dependence and assume that mortality rate depends on MUAC-Z in the same way as on WAZ (see Table 2 in [9] for a justification of this extrapolation), and set $a = -7.13, b = 0.722$.

We also assume that MUAC-Z for children in any given month has a normal distribution. If the mean and variance of the distribution are μ and σ, the expected number of child deaths in the population of N children in this month is

$$
\begin{aligned}
D &= E[Ne^{a-bZ}], && (16)\\
&= N\int_{-\infty}^{\infty} \frac{1}{\sqrt{2\pi}} e^{a-b(\mu+\sigma x)} e^{-\frac{x^2}{2}}\, dx, && (17)\\
&= Ne^{a+\frac{b^2\sigma^2}{2}-b\mu}. && (18)
\end{aligned}
$$

If we only have a forecast $f = \mu + \epsilon$ of the mean MUAC-Z, where $\epsilon \sim \mathcal{N}(0, \sigma_\epsilon^2)$, then the expected number of deaths conditional on the forecast is

$$
\begin{aligned}
D &= E[E[Ne^{a-bZ}|\epsilon]], && (19)\\
&= E[Ne^{a+\frac{b^2\sigma^2}{2}-b(f-\epsilon)}], && (20)\\
&= Ne^{a+\frac{b^2(\sigma^2+\sigma_\epsilon^2)}{2}-bf}. && (21)
\end{aligned}
$$

C.2 The Exact Solution With a Single Shipment Mode

In this subsection, we consider the simplified problem that has one shipment mode and allows negative food shipments. In our problem formulation, we make the following 11 assumptions:

1. There is a single delivery mode with a lead time of L months;
2. There is a single community with N children;
3. The MUAC-Z among children in the community in month t has a normal distribution with mean μ_t and standard deviation σ;
4. By (8), the mean MUAC-Z in month t in the absence of food assistance is $\tilde{\mu}_{t\text{mod}12} + \sum_{\tau=0}^{H} \epsilon_{t-\tau,t}$, where $\tilde{\mu}_{t\text{mod}12}$ is the long-term mean MUAC-Z in month t in the absence of food assistance, $\epsilon_{t-\tau,t}$ is the forecast update for month t made in month $t-\tau$, and $H = 9$ mo is the forecast horizon. Similarly, the forecast for month t made in month $t-k$ is $f_{t-k,t} = \mu_{t\text{mod}12} + \sum_{\tau=k}^{H} \epsilon_{t-\tau,t}$;
5. The mean MUAC-Z in month t depends linearly on the food assistance delivered in the last 54 months (see §A.2) via

$$\mu_t = \tilde{\mu}_{t\text{mod}12} + \sum_{\tau=0}^{H} \epsilon_{t-\tau,t} + \frac{d}{N} \sum_{\tau=0}^{53} \frac{54-\tau}{54} O_{t-\tau-L}, \qquad (22)$$

   where $d = 0.015$/kg is the impact of food assistance on the mean MUAC-Z, N is the

number of children, and O_t is the order placed in month t;

6. The vector of forecast updates, $(\epsilon_{t,t}, \epsilon_{t,t+1}, \ldots, \epsilon_{t,t+H})$ has a multivariate normal distribution with mean zero and the covariance structure described in § B:

$$Cov(\epsilon_{t-\tau_1,t+\tau_2}, \epsilon_{t-\tau_3,t+\tau_4}) = \begin{cases} \Sigma^1_{\tau_1+\tau_2,\tau_3+\tau_4} & \text{if } \tau_2 = \tau_4; \\ \Sigma^2_{\tau_1+\tau_2,\tau_3+\tau_4} & \text{if } \tau_1 = \tau_3; \\ 0 & \text{otherwise}; \end{cases} \tag{23}$$

7. The standard deviation of MUAC-Z in each month, $\sigma = 0.62$, is assumed to be known in advance;

8. Month $T = 120$ is the last month that we can place an order, and month $T + L$ – when that order is delivered – is the last period that we include in the objective function;

9. Orders are allowed to be negative, which corresponds to the collection of food from the communities to increase the available budget and enable larger food shipments in future months;

10. The objective is to minimize the expected number of child deaths (as given in (8)) during months $1, \ldots, T + L$;

11. An annual budget B is made available in months $t = 1, 13, 25, \ldots$. Each order decreases the available budget according to

$$B_{t+1} = B_t - cO_t, \tag{24}$$

where B_t is the budget available in month t, c is the cost per kg of food assistance, and we assume that the remaining budget is used in the final month of each fiscal year.

This problem can be formulated and solved as a dynamic program [10]. In month t, the decision is the order quantity O_t and the system state of the dynamic program consists of all

previous orders $(O_{t-54},\ldots,O_{t-1})$, all $(H+1)(H+2)/2$ forecast updates observed up to month t that are for the current or future months $(\epsilon_{t-H,t};\epsilon_{t-H+1,t},\epsilon_{t-H+1,t+1};\ldots;\epsilon_{t-1,t},\epsilon_{t-1,t+1},\ldots,\epsilon_{t-1,t+H-1};\epsilon_{t,t},\epsilon_{t,t+1},\ldots,\epsilon_{t,t+H})$, and the available budget B_t. Because the order quantity O_t impacts child mortality only in months $t+L,\ldots,T+L$, the optimal expected cost-to-go in month t, denoted by J_t, represents the cost starting from month $t+L$, and the expectation evaluated in month t, denoted by $E_t[\cdot]$, is with respect to the forecast updates made in month $t+1$ $(\epsilon_{t+1,t+1},\ldots,\epsilon_{t+1,t+1+H})$. The solution to this dynamic program is given in the following theorem.

Theorem 1. *The optimal expected cost-to-go in month t is the exponent of an affine function (with parameters* $(\alpha^{(t)},\beta_\tau^{(t)},\gamma_{\tau_1,\tau_2}^{(t)},\delta^{(t)})$*) of the state variables:* $J_t=\exp(\alpha^{(t)}+\sum_{\tau=1}^{53}\beta_\tau^{(t)}O_{t-\tau}+\sum_{\tau_1=0}^{H}\sum_{\tau_2=0}^{H-\tau_1}\gamma_{\tau_1,\tau_2}^{(t)}\epsilon_{t-\tau_1,t+\tau_2}+\delta^{(t)}B_t)$*. The optimal order in month t is an affine function (with parameters* $(\nu^{(t)},\eta_\tau^{(t)},\theta_{\tau_1,\tau_2}^{(t)},\lambda^{(t)})$*) of the state variables:* $O_t^*=\nu^{(t)}+\sum_{\tau=1}^{H}\eta_\tau^{(t)}O_{t-\tau}+\sum_{\tau_1=0}^{H}\sum_{\tau_2=0}^{H-\tau_1}\theta_{\tau_1,\tau_2}^{(t)}\epsilon_{t-\tau_1,t+\tau_2}+\lambda^{(t)}B_t$.

Proof. We prove the theorem by backward induction, starting with month T, which is the last month of its year (i.e., T mod 12=0). Hence, the entire available budget is used; i.e., $O_T^*=\frac{B_T}{c}$. The decision O_T^* affects child mortality only in month $T+L$, yielding

$$\begin{aligned} J_T &= E_T[N\exp(a+\frac{b^2\sigma_{T+L}^2}{2}-b(\mu_{T+L}+\sum_{i=0}^{H}\epsilon_{T+L-i,T+L} \\ &\quad +\frac{d}{N}\sum_{\tau=1}^{53}\frac{54-\tau}{54}O_{T-\tau}+\frac{dB_T}{cN}))] \text{ by (20), (22),} && (25) \\ &= N\exp(a+\frac{b^2(\sigma_{T+L}^2+\sum_{i=0}^{L-1}\sum_{j=0}^{L-1}\Sigma_{ij}^1)}{2}-b(\mu_{T+L}+\sum_{i=L}^{H}\epsilon_{T+L-i,T+L} \\ &\quad +\frac{d}{N}\sum_{\tau=1}^{53}\frac{54-\tau}{54}O_{T-\tau}+\frac{dB_T}{cN})) \text{ by (21), (23).} && (26) \end{aligned}$$

Equation (26) proves the base of induction and establishes the coefficients for $t = T$:

$$\alpha^{(T)} = \ln N + a + \frac{b^2(\sigma^2_{T+L} + \sum_{i=0}^{L-1}\sum_{j=0}^{L-1}\Sigma^1_{ij})}{2} - b\mu_{T+L}, \tag{27}$$

$$\beta^{(T)}_\tau = -\frac{bd}{N}\frac{54-\tau}{54}, \tag{28}$$

$$\gamma^{(T)}_{\tau_1,\tau_2} = \begin{cases} -b & \text{if } \tau_2 = L,\ 0 \le \tau_1 \le H - L; \\ 0 & \text{otherwise,} \end{cases} \tag{29}$$

$$\delta^{(T)} = -\frac{bd}{cN}, \tag{30}$$

$$\nu^{(T)} = 0, \tag{31}$$

$$\eta^{(T)}_\tau = 0 \ \ \forall\tau, \tag{32}$$

$$\theta^{(T)}_{\tau_1,\tau_2} = 0 \ \ \forall\tau, \tag{33}$$

$$\lambda^{(T)} = \frac{1}{c}. \tag{34}$$

Next, we suppose that the relationships in (26)-(34) hold in months $t+1, ..., T$, and prove that they hold in month t. We let C_t be the child mortality rate in month t, and note that the dynamic programming recursion dictates that the optimal order quantity O_t minimizes $E_t[C_{t+L} + J_{t+1}]$ subject to the budget equation in (24), where

$$E_t[C_{t+L}] = N\exp(a + \frac{b^2(\sigma^2_{t+L} + \sum_{i=0}^{L-1}\sum_{j=0}^{L-1}\Sigma^1_{ij})}{2} - b(\mu_{t+L} + \sum_{i=L}^{H}\epsilon_{t+L-i,t+L} + \frac{d}{N}\sum_{\tau=0}^{53}\frac{54-\tau}{54}O_{t-\tau})), \tag{35}$$

$$\begin{aligned} E_t[J_{t+1}] &= E_t[\exp(\alpha^{(t+1)} + \sum_{\tau=1}^{53}\beta^{(t+1)}_\tau O_{t+1-\tau} \\ &+ \sum_{\tau_1=0}^{H}\sum_{\tau_2=0}^{H-\tau_1}\gamma^{(t+1)}_{\tau_1,\tau_2}\epsilon_{t+1-\tau_1,t+1+\tau_2} + \delta^{(t+1)}B_{t+1})], &(36)\\ &= \exp(\alpha^{(t+1)} + \sum_{\tau=1}^{52}\beta^{(t+1)}_{\tau+1}O_{t-\tau} + \sum_{\tau_1=1}^{H}\sum_{\tau_2=0}^{H-\tau_1}\gamma^{(t+1)}_{\tau_1,\tau_2}\epsilon_{t+1-\tau_1,t+1+\tau_2} + \delta^{(t+1)}(B_t - cO_t)) \\ &\cdot e^{\beta^{(t+1)}_1 O_t}E_t[\exp(\sum_{\tau_2=0}^{H}\gamma^{(t+1)}_{0,\tau_2}\epsilon_{t+1,t+1+\tau_2})] \ \text{ by } \ (24). &(37)\end{aligned}$$

We define the functions

$$A_{t+L} = N\exp(a+\frac{b^2(\sigma^2_{t+L}+\sum_{i=0}^{L-1}\sum_{j=0}^{L-1}\Sigma^1_{ij})}{2} - b(\mu_{t+L}+\sum_{i=L}^{H}\epsilon_{t+L-i,t+L}+\frac{d}{N}\sum_{\tau=1}^{53}\frac{54-\tau}{54}O_{t-\tau})), \tag{38}$$

$$\begin{aligned} A_{t+1} &= \exp(\alpha^{(t+1)}+\sum_{\tau=1}^{52}\beta^{(t+1)}_{\tau+1}O_{t-\tau}+\sum_{\tau_1=1}^{H}\sum_{\tau_2=0}^{H-\tau_1}\gamma^{(t+1)}_{\tau_1,\tau_2}\epsilon_{t+1-\tau_1,t+1+\tau_2}+\delta^{(t+1)}B_t) \\ &\cdot E_t[\exp(\sum_{\tau_2=0}^{H}\gamma^{(t+1)}_{0,\tau_2}\epsilon_{t+1,t+1+\tau_2})], \end{aligned} \tag{39}$$

so that equations (35) and (37) can be re-expressed as

$$E_t[C_{t+L}] = A_{t+L}e^{-bdO_t/N}, \tag{40}$$

$$E_t[J_{t+1}] = A_{t+1}e^{(\beta^{(t+1)}_1 - c\delta^{(t+1)})O_t}. \tag{41}$$

Note that A_{t+L} and A_{t+1} are functions of $O_{t-53},\ldots,O_{t-1}$ and $\epsilon_{t-\tau_1,t+\tau_2}$ for $1\le\tau_1\le H,\ 0\le\tau_2\le H-\tau_1$, but not O_t or $\epsilon_{t,t+\tau_2}$.

Before solving $\min_{O_t} E_t[C_{t+L}]+E_t[J_{t+1}]$, we simplify A_{t+1} in (39). The expectation in (39) is conditional on the relevant forecast updates which are available in period t, which we denote by $\{\epsilon\}_t = \{\epsilon_{t-\tau_1,t+\tau_2}$ for $0\le\tau_1\le H,\ 0\le\tau_2\le H-\tau_1\}$. Because $E[\exp(X)] = \exp(\mu+\frac{\sigma^2}{2})$ for a random variable $X\sim\mathcal{N}(\mu,\sigma^2)$, we need to find the conditional mean and variance of $\epsilon_{t+1,t+1+\tau_2}$ in (39), given $\{\epsilon\}_t$. These forecast updates have a multivariate normal distribution, and hence the conditional mean is linear in the variables that we condition on:

$$E[\epsilon_{t+1,t+1+\tau}|\{\epsilon\}_t] = \sum_{\tau_1=0}^{H}\sum_{\tau_2=0}^{H-\tau_1}p^{\tau}_{\tau_1,\tau_2}\epsilon_{t-\tau_1,t+\tau_2}. \tag{42}$$

For a given τ, the vector of coefficients $p^{\tau}_{\tau_1,\tau_2}$ in (42) can be expressed via the covariance

matrices Σ^1, Σ^2 in (23) as follows:

$$p^\tau = \Sigma_{12}^\tau \Sigma_{22}^{-1}, \tag{43}$$

where Σ_{12}^τ is a vector of covariances of the random variable $\epsilon_{t+1,t+1+\tau}$ with the vector $\{\epsilon\}_t$, and Σ_{22} is the covariance matrix of the vector $\{\epsilon\}_t$. Denoting the variance of $(\sum_{\tau_2=0}^{H} \gamma_{0,\tau_2}^{(t+1)} \epsilon_{t+1,t+1+\tau_2} | \{\epsilon\}_t)$ by $V(\gamma^{(t+1)})$, we can express the expectation in (39) as

$$
\begin{aligned}
E_t[\exp(\sum_{\tau_2=0}^{H} \gamma_{0,\tau_2}^{(t+1)} \epsilon_{t+1,t+1+\tau_2})] &= \exp(\sum_{\tau_2=0}^{H} \gamma_{0,\tau_2}^{(t+1)} E[\epsilon_{t+1,t+1+\tau_2} | \{\epsilon\}_t] + \frac{V(\gamma^{(t+1)})}{2}), && (44) \\
&= \exp(\sum_{\tau=0}^{H} \gamma_{0,\tau}^{(t+1)} \sum_{\tau_1=0}^{H} \sum_{\tau_2=0}^{H-\tau_1} p_{\tau_1,\tau_2}^{\tau} \epsilon_{t-\tau_1,t+\tau_2} | \{\epsilon\}_t] \\
&\quad + \frac{V(\gamma^{(t+1)})}{2}) \text{ by (42)}, && (45) \\
&= \exp(\sum_{\tau_1=0}^{H} \sum_{\tau_2=0}^{H-\tau_1} \epsilon_{t-\tau_1,t+\tau_2} \sum_{\tau=0}^{H} \gamma_{0,\tau}^{(t+1)} p_{\tau_1,\tau_2}^{\tau} | \{\epsilon\}_t] + \frac{V(\gamma^{(t+1)})}{2}) && (46)
\end{aligned}
$$

Moreover, using the change of variables $t_1 = \tau_1 - 1$ and $t_2 = \tau_2 + 1$ and letting $I_{\{x\}}$ denote the indicator function of x, we can write the double summation in (39) as

$$
\begin{aligned}
\sum_{\tau_1=1}^{H} \sum_{\tau_2=0}^{H-\tau_1} \gamma_{\tau_1,\tau_2}^{(t+1)} \epsilon_{t+1-\tau_1,t+1+\tau_2} &= \sum_{t_1=0}^{H-1} \sum_{t_2=1}^{H-t_1} \gamma_{t_1+1,t_2-1}^{(t+1)} \epsilon_{t-t_1,t+t_2}, && (47) \\
&= \sum_{\tau_1=0}^{H} \sum_{\tau_2=0}^{H-\tau_1} I_{\{\tau_1<H,\tau_2>1\}} \gamma_{\tau_1+1,\tau_2-1}^{(t+1)} \epsilon_{t-\tau_1,t+\tau_2}. && (48)
\end{aligned}
$$

Using (46) and (48), we can re-express (39) as

$$
\begin{aligned}
A_{t+1} &= \exp(\alpha^{(t+1)} + \sum_{\tau=1}^{52} \beta_{\tau+1}^{(t+1)} O_{t-\tau} + \sum_{\tau_1=0}^{H-1} \sum_{\tau_2=1}^{H-\tau_1} (\gamma_{\tau_1+1,\tau_2-1}^{(t+1)} I_{\{\tau_1<H,\tau_2>1\}} && (49) \\
&\quad + \sum_{\tau=0}^{H} p_{\tau_1,\tau_2}^{\tau} \gamma_{0,\tau_2}^{(t+1)}) \epsilon_{t-\tau_1,t+\tau_2} + \delta^{(t+1)} B_t - \frac{V(\gamma^{(t+1)})}{2}). && (50)
\end{aligned}
$$

By (40)-(41), the optimization problem is

$$\min_{O_t} \; A_{t+L} e^{-bdO_t/N} + A_{t+1} e^{(\beta_1^{(t+1)} - c\delta^{(t+1)})O_t}, \tag{51}$$

which is convex. If $\beta_1^{(t+1)} - c\delta^{(t+1)} > 0$ (we prove later that this is indeed the case), this problem has a unique minimum defined by the first-order condition

$$-A_{t+L} e^{-bdO_t/N} + (\beta_1^{(t+1)} - c\delta^{(t+1)}) A_{t+1} e^{(\beta_1^{(t+1)} - c\delta^{(t+1)})O_t} = 0. \tag{52}$$

The solution to (52) is

$$O_t^* = \frac{1}{\frac{bd}{N} + \beta_1^{(t+1)} - c\delta^{(t+1)}} \ln \frac{A_{t+L}}{(\beta_1^{(t+1)} - c\delta^{(t+1)}) A_{t+1}}, \tag{53}$$

which, after substituting in A_{t+L} and A_{t+1} from (38) and (49), is an affine function of $\{\epsilon\}_t, B_t, O_{t-53}, \ldots, O_{t-1}$ with parameters given by

$$\nu^{(t)} = \frac{\left(-\ln(\beta_1^{(t+1)} - c\delta^{(t+1)}) + a + \ln N + \frac{b^2(\sigma_{t+L}^2 + \sum_{i=0}^{L-1}\sum_{j=0}^{L-1}\Sigma_{ij}^1)}{2} - b\mu_{t+L} - \alpha^{(t+1)} + \frac{V(\gamma^{(t+1)})}{2}\right)}{\frac{bd}{N} + \beta_1^{(t+1)} - c\delta^{(t+1)}}, \tag{54}$$

$$\eta_\tau^{(t)} = \frac{\frac{54-\tau}{54}\frac{bd}{N} - \beta_{\tau+1}^{(t+1)} I_{\{\tau<53\}}}{\frac{bd}{N} + \beta_1^{(t+1)} - c\delta^{(t+1)}}, \tag{55}$$

$$\theta_{\tau_1,\tau_2}^{(t)} = \frac{-bI_{\{0\le\tau_1\le H-L,\tau_2=L\}} - \gamma_{\tau_1+1,\tau_2-1}^{(t+1)} I_{\{\tau_1<H,\tau_2>1\}} - \sum_{\tau=0}^{H} p_{\tau_1,\tau_2}^{\tau}\gamma_{0,\tau_2}^{(t+1)}}{\frac{bd}{N} + \beta_1^{(t+1)} - c\delta^{(t+1)}}, \tag{56}$$

$$\lambda^{(t)} = -\frac{\delta^{(t+1)}}{\frac{bd}{N} + \beta_1^{(t+1)} - c\delta^{(t+1)}}. \tag{57}$$

Substituting (53) into (51), we get the optimal cost-to-go starting from period t,

$$J_t = A_{t+L}^{\frac{\beta_1^{(t+1)} - c\delta^{(t+1)}}{\frac{bd}{N} + \beta_1^{(t+1)} - c\delta^{(t+1)}}} A_{t+1}^{\frac{\frac{bd}{N}}{\frac{bd}{N} + \beta_1^{(t+1)} - c\delta^{(t+1)}}} \cdot \left(\left(\beta_1^{(t+1)} - c\delta^{(t+1)}\right)^{\frac{\frac{bd}{N}}{\frac{bd}{N} + \beta_1^{(t+1)} - c\delta^{(t+1)}}} + \left(\beta_1^{(t+1)} - c\delta^{(t+1)}\right)^{-\frac{\beta_1^{(t+1)} - c\delta^{(t+1)}}{\frac{bd}{N} + \beta_1^{(t+1)} - c\delta^{(t+1)}}} \right). \tag{58}$$

Because A_{t+L} and A_{t+1} are both exponentials of affine functions of $\{\epsilon\}_t, B_t, O_{t-53}, \ldots, O_{t-1}$, the cost-to-go function J_t is also an exponential of an affine function with the following parameters:

$$\begin{aligned}
\alpha^{(t)} &= \ln\left(\left(\beta_1^{(t+1)} - c\delta^{(t+1)}\right)^{\frac{\frac{bd}{N}}{\frac{bd}{N}+\beta_1^{(t+1)}-c\delta^{(t+1)}}} + \left(\beta_1^{(t+1)} - c\delta^{(t+1)}\right)^{-\frac{\beta_1^{(t+1)}-c\delta^{(t+1)}}{\frac{bd}{N}+\beta_1^{(t+1)}-c\delta^{(t+1)}}}\right) \\
&\quad + \frac{\beta_1^{(t+1)} - c\delta^{(t+1)}}{\frac{bd}{N} + \beta_1^{(t+1)} - c\delta^{(t+1)}}\left(\ln N + a + \frac{b^2(\sigma_{t+L}^2 + \sum_{i=0}^{L-1}\sum_{j=0}^{L-1}\Sigma_{ij}^1)}{2} - b\mu_{t+L}\right) \\
&\quad + \frac{\frac{bd}{N}}{\frac{bd}{N} + \beta_1^{(t+1)} - c\delta^{(t+1)}}\left(\alpha^{(t+1)} - \frac{V(\gamma^{(t+1)})}{2}\right), &(59)\\
\beta_\tau^{(t)} &= -\frac{\beta_1^{(t+1)} - c\delta^{(t+1)}}{\frac{bd}{N} + \beta_1^{(t+1)} - c\delta^{(t+1)}}\frac{54-\tau}{54}\frac{bd}{N} + \frac{\frac{bd}{N}}{\frac{bd}{N} + \beta_1^{(t+1)} - c\delta^{(t+1)}}\beta_{\tau+1}^{(t+1)} I_{\{\tau<53\}}, &(60)\\
\gamma_{\tau_1,\tau_2}^{(t)} &= -\frac{\beta_1^{(t+1)} - c\delta^{(t+1)}}{\frac{bd}{N} + \beta_1^{(t+1)} - c\delta^{(t+1)}} bI_{\{0\le\tau_1\le H-L,\tau_2=L\}} \\
&\quad + \frac{\frac{bd}{N}}{\frac{bd}{N} + \beta_1^{(t+1)} - c\delta^{(t+1)}}\left(\gamma_{\tau_1+1,\tau_2-1}^{(t+1)} I_{\{\tau_1<H,\tau_2>1\}} + \sum_{\tau=0}^{H} p_{\tau_1,\tau_2}^{\tau}\gamma_{0,\tau_2}^{(t+1)}\right), &(61)\\
\delta^{(t)} &= \frac{\frac{bd}{N}}{\frac{bd}{N} + \beta_1^{(t+1)} - c\delta^{(t+1)}}\delta^{(t+1)}. &(62)
\end{aligned}$$

We complete the proof by showing that $\beta_1^{(t+1)} - c\delta^{(t+1)} > 0$, which was needed to derive (52). We do this by showing that both $\beta_1^{(t)} - c\delta^{(t)}$ and $\beta_\tau^{(t)} - \beta_{\tau-1}^{(t)}$ equal $bd/(54N)$ for all t. Using backward induction beginning with $t = T$, we employ equation (26) to compute

$$\begin{aligned}
\beta_1^{(T)} - c\delta^{(T)} &= -\frac{bd}{N}\frac{53}{54} + \frac{bd}{N}, &(63)\\
&= \frac{bd}{54N} > 0, &(64)\\
\beta_\tau^{(T)} - \beta_{\tau-1}^{(T)} &= -\frac{bd}{N}\frac{54-\tau}{54} + \frac{bd}{N}\frac{54-\tau+1}{54}, &(65)\\
&= \frac{bd}{54N}. &(66)
\end{aligned}$$

Assuming (64) and (66) hold for month $t+1$, we now show that they hold for month

t. We have

$$\beta_\tau^{(t)} - \beta_{\tau-1}^{(t)} = \frac{\beta_1^{(t+1)} - c\delta^{(t+1)}}{\frac{bd}{N} + \beta_1^{(t+1)} - c\delta^{(t+1)}} \frac{1}{54}\frac{bd}{N} + \frac{\frac{bd}{N}}{\frac{bd}{N} + \beta_1^{(t+1)} - c\delta^{(t+1)}} (\beta_{\tau+1}^{(t+1)} - \beta_\tau^{(t+1)}) \text{ by (60)}, \tag{67}$$

$$= \frac{bd}{54N} \text{ by (64), (66).} \tag{68}$$

Similarly,

$$\beta_1^{(t)} - c\delta^{(t)} = -\frac{\beta_1^{(t+1)} - c\delta^{(t+1)}}{\frac{bd}{N} + \beta_1^{(t+1)} - c\delta^{(t+1)}} \frac{53}{54}\frac{bd}{N} + \frac{\frac{bd}{N}}{\frac{bd}{N} + \beta_1^{(t+1)} - c\delta^{(t+1)}} \left(\beta_2^{(t+1)} - c\delta^{(t+1)}\right) \text{ by (60), (62)}, \tag{69}$$

$$= -\frac{1}{55}\frac{53}{54}\frac{bd}{N} + \frac{54}{55}(\beta_2^{(t+1)} - c\delta^{(t+1)}) \text{ by (64)}, \tag{70}$$

$$= -\frac{1}{55}\frac{53}{54}\frac{bd}{N} + \frac{54}{55}(\beta_2^{(t+1)} - \beta_1^{(t+1)} + \beta_1^{(t+1)} - c\delta^{(t+1)}), \tag{71}$$

$$= -\frac{1}{55}\frac{53}{54}\frac{bd}{N} + \frac{54}{55}\frac{2bd}{54N} \text{ by (64), (66)}, \tag{72}$$

$$= \frac{bd}{54N}. \tag{73}$$

□

C.3 Numerical optimization

The model described in §C.2 is based on two unrealistic assumptions: single delivery mode and negative orders. Because we were not able to obtain a closed-form solution without these assumptions and because the original problem (i.e., with two delivery modes and nonnegative orders) is unwieldy, we resort in this subsection to a suboptimal approach to the original problem. More specifically, motivated by Theorem 1, we numerically find the death-minimizing policy among all ordering policies that are affine in the state variables.

To allow for two delivery modes with nonnegative orders, we modify Assumptions 1, 4, 9

and 11 in §C.2:

1′. There are two delivery modes: a fast mode with a lead time of L_f months and a slow mode with a lead time of L_s months;

4′. The mean MUAC-Z in month t depends linearly on the food assistance delivered in the last 54 months (see §A.2) via

$$\mu_t = \mu_t^0 + \frac{d}{N} \sum_{\tau=0}^{53} \frac{54-\tau}{54} (O^f_{t-\tau-L_f} + O^s_{t-\tau-L_s}), \tag{74}$$

where μ_t^0 is the mean MUAC-Z in month t in the absence of food assistance, O_t^f and O_t^s are the orders placed by the fast and slow shipment modes in month t, and $d = 0.015/\text{kg}$ denotes the impact of food assistance on the mean MUAC-Z;

9′. Orders must be nonnegative;

11′. Fast and slow delivery modes have separate annual budgets ($B_f = \frac{c_f l}{c_f l + c_s(1-l)} B$ and $B_s = \frac{c_s(1-l)}{c_f l + c_s(1-l)} B$) that are made available in months $t = 1, 13, 25, \ldots$, where $t = 1$ corresponds to October, which is the beginning of the fiscal year. The remaining budgets available from month t until the end of the year, B_t^f and B_t^s, evolve according to

$$B^f_{t+1} = B^f_t - c_f O^f_t, \tag{75}$$

$$B^s_{t+1} = B^s_t - c_s O^s_t, \tag{76}$$

where c_f and c_s are the cost per kg of food assistance under the fast and slow shipment modes.

Hence, the decisions in month t are (O_t^f, O_t^s), and in the system state we replace $(O_{t-54}, \ldots, O_{t-1})$ by $(O^f_{t-54}, \ldots, O^f_{t-1}, O^s_{t-54}, \ldots, O^s_{t-1})$. Because of the risk of overfitting, we do not allow are decisions to be general affine functions of the state as in Theorem 1. The affine policy that we consider requires the order quantity for each mode ($O_t^m, m = \{f, s\}$) to have an intercept term that is independent of the month, to depend on previous orders only

through their weighted linear combination $\sum_{\tau=1}^{53} O_{t-\tau}^m \frac{54-\tau}{54}$, to depend on forecast updates only through forecasts for future months, to have the coefficients of the available budget and the forecasts be exponential functions of the month of the year and the forecast lead time. Imposing these restrictions has allowed us to improve the out-of-sample performance of our policies. Adapting the relevant notation from Theorem 1, we search for the best policy from the affine parametric class defined by

$$O_t^m = \nu_m + e^{\lambda_0^m + \lambda_1^m t} B_t^m - \sum_{\tau=L_m}^{H} e^{\theta_0^m - \theta_1^m \tau} f_{t,t+\tau} + \eta_m \sum_{\tau=1}^{53} O_{t-\tau}^m \frac{54-\tau}{54} \quad \text{for } m = \{f, s\}. \qquad (77)$$

We evaluate the cost of a given policy, defined by the 12 parameters ($\nu_f, \lambda_0^f, \lambda_1^f, \theta_0^f, \theta_1^f, \eta_f$, $\nu_s, \lambda_0^s, \lambda_1^s, \theta_0^s, \theta_1^s, \eta_s$), by generating a forecast evolution for 120 months according to the covariance structure of the forecast updates, and computing orders according to the policy under consideration. To remove the impact of stochasticity, the same generated sequence of forecasts is used to evaluate the costs for different parameter values. Because orders placed in the previous 53 months impact the current value of the mean MUAC-Z, we have to wait at least 54 months (we actually wait 72 months) before recording the costs to ensure that the cost is indeed determined by the orders placed according to the policy under consideration and not orders placed before the beginning of the simulation period (which we assume to be placed by allocating annual budgets equally among 12 months regardless of state variables). The direct application of (77) could result in negative orders or orders larger than the available budgets. In such cases, no order is placed or the entire budget is spent, respectively.

Optimal values of the 12 parameters in (77) are searched for numerically by the Nelder-Mead algorithm [11], which is a robust algorithm that can deal with objective functions that lack smoothness. Because this algorithm finds only a local minimum and our objective function is non-convex, we use multiple random restarts from multiple (in our case, 300) points around the starting point, with each component of the parameter vector randomly changed by up to 20%. We use starting values inspired by Theorem 1, except for η_f and η_s,

which are optimized conditional on the remainder of the initial values:

$$\nu_m = \frac{\sum_{t=1}^{12} \sum_{\tau=0}^{H-L_m} e^{\beta_0^m - \beta_1^m \tau} \mu_t}{12} - \eta_m B_m \frac{53}{2(12)}, \tag{78}$$

$$\lambda_0^m = -12 \ln(\frac{55}{54}), \tag{79}$$

$$\lambda_1^m = \ln(\frac{54}{55}), \tag{80}$$

$$\theta_0^m = -\ln 55 + (H+1) \ln(\frac{55}{54}), \tag{81}$$

$$\theta_1^m = -\ln(\frac{55}{54}), \tag{82}$$

$$\eta_m = -0.007. \tag{83}$$

References

[1] Mude, A. G., Barrett, C. B., McPeak, J. G., Kaitho, R., Kristjanson, P. Empirical forecasting of slow-onset disasters for improved emergency response: An application to Kenya's arid north. *Food Policy* **34**, 329-339, 2009.

[2] World Food Programme. Food aid information system. Accessed at http://www.wfp.org/fais/reports/irma-by-food-aid-type/run/2012/All/All/Kenya/All on April 10, 2015.

[3] Isanaka, S., Nombela, N., Djibo, A., Poupard, M., Van Beckhoven, D., Gaboulaud, V., Guerin, P.J., Grais, R.F. Effect of preventive supplementation with ready-to-use therapeutic food on the nutritional status, mortality and morbidity of children aged 6 to 60 months in Niger. *JAMA* **301**, 277-285, 2009.

[4] Zou, H., Hastie, T. Regularization and variable selection via the elastic net. *Journal of the Royal statistical Society: Series B (Statistical Methodology)* **67**, 301-320, 2005.

[5] Heath, D. C., Jackson, P. L. Modeling the evolution of demand forecasts with application to safety stock analysis in production/distribution systems. *IIE Transactions* **26**, 17-30,

1994.

[6] Fan, J., Fan, Y., Lv, J. High dimensional covariance matrix estimation using a factor model. *J. Econometrics* **147**, 186-197, 2008.

[7] Chen, Y., Wiesel, A., Eldar, Y. C., Hero, A. O. Shrinkage algorithms for MMSE covariance estimation. *IEEE Transactions on Signal Processing* **58**, 5016-5029, 2010.

[8] Fishman, S. M., Caulfield, L. E., de Onis, M., Blossner, M., Hyder, A. A., Mullany, L., Black, R. E. Childhood and maternal underweight. Chapter 2 of *Comparative quantification of health risks: global and regional burden of disease attributable to selected major risk factors, Vol. 1.* Eds, Ezzati, M., Lopez, A. D., Rodgers, A., Murray, C. J. L. World Health Organization, Geneva, 2004.

[9] Garenne, M., Maire, B., Fontaine, O., Briend, A. Distributions of mortality risk attributable to low nutritional status in Niakhar, Senegal. *J. Nutrition* **136**, 2893-2900, 2006.

[10] Bertsekas, D. P. *Dynamic programming and stochastic control.* Academic Press, New York, 1976.

[11] Avriel, M. *Nonlinear programming: analysis and methods.* Prentice-Hall, Englewood Cliffs, NJ, 1976.

Community code	Community name	w_i
10404	Kaeris	0.051
10606	Letea	0.035
10707	Lopur	0.035
10808	Loima	0.013
10909	Naremit	0.036
11011	Napeililim	0.02
11112	Loboolo	0.024
11113	Kalimapus	0.027
11214	Kerio	0.02
11315	Lokapel	0.016
11416	Kalemungorok	0.024
11517	Napusimoru	0.014
11518	Kalapata	0.043
11619	Lokwamosing	0.014
11620	Katilia	0.015
11721	Napeitom	0.022
20109	Maron	0.022
20110	Nakoko	0.035
20203	Kapunyany	0.026
20206	Kolloa	0.021
20208	Loiwat	0.021
20305	Kinyach	0.025
20412	Yatya	0.013
30103	Karare	0.026
30110	Sagante	0.036
30205	Loglogo	0.013
30208	Ngurnit	0.023
30306	Loiyangalani	0.013
30402	Kalacha	0.038
30407	Maikona	0.019
30509	North-Horr	0.024
40113	Nachola	0.022
40212	Loosuk	0.024
40402	Arsim	0.035
40403	Kawop	0.023
40418	South Horr	0.026
40420	Tuum	0.036
40511	Lodungokwe	0.012
40519	Swaari	0.011
40605	Kiltamany	0.02
40607	Laresoro	0.025

Table A: The analyzed communities, along with their child populations and the proportion of the total child population from their community (w_i).

Group	Time Lags	Variables	Number of Variables
MUAC moments	1,2,3	MUAC mean, std. dev., skewness, kurtosis	12
herd dynamics (large stock)	1,2	size, mortality, sales, slaughters	8
herd dynamics (small stock)	1,2	size, mortality, sales, slaughters	8
food aid	1,2	Unimix kgs, Unimix fraction, regular kgs, regular fraction	8
biophysical	2,3,4,5,6	rain, rain^2, forage, forage^2, NDVI, NDVI^2	30

Table B: The explanatory variables X_{ijt} in the forecasting model (1) for a fixed i and t, for $j = 1, \ldots, 66$. Under food aid, Unimix is a corn-soy blend, kgs refers to the number of kg received per recipient household, fraction refers to the proportion of households that received the food, and regular refers to regular cereals. NDVI is the normalized differenced vegetation idex, which is a measure of the relative density of green vegetation within an area.

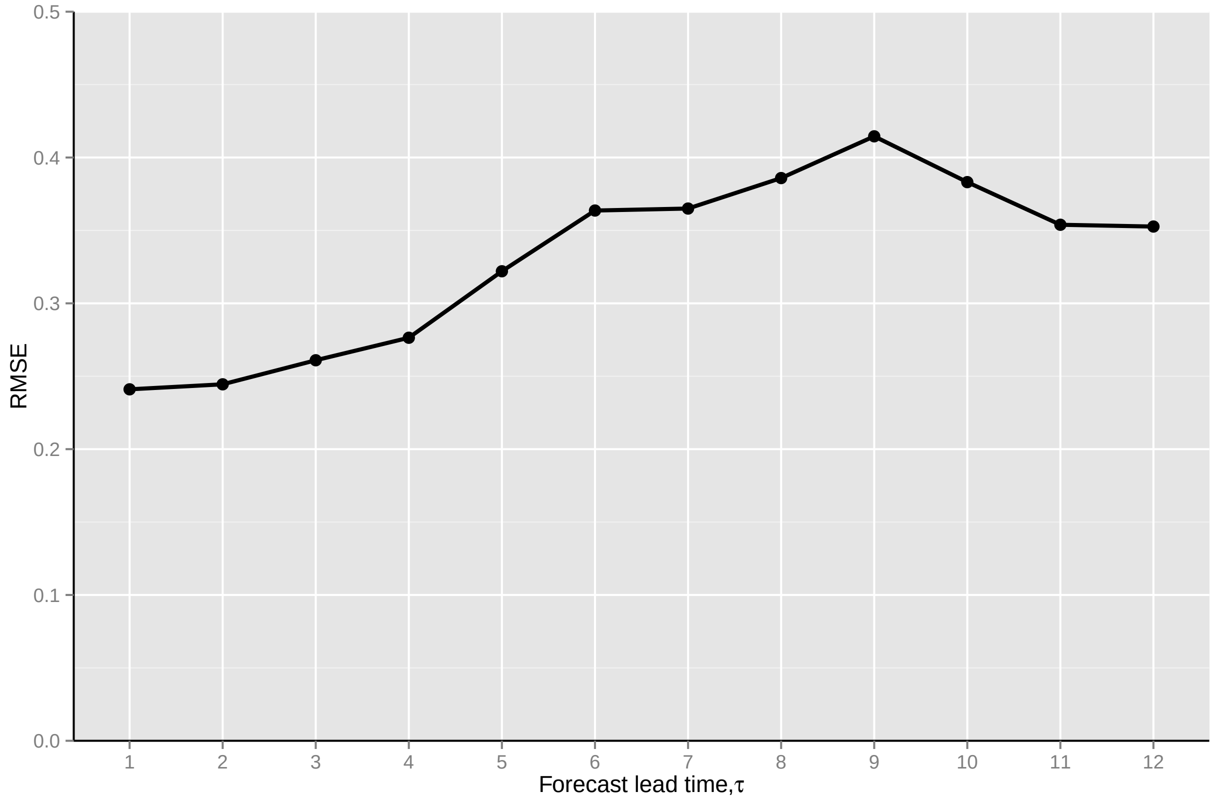

Fig A: The forecast root mean square error (RMSE) as a function of the forecast lead time τ.

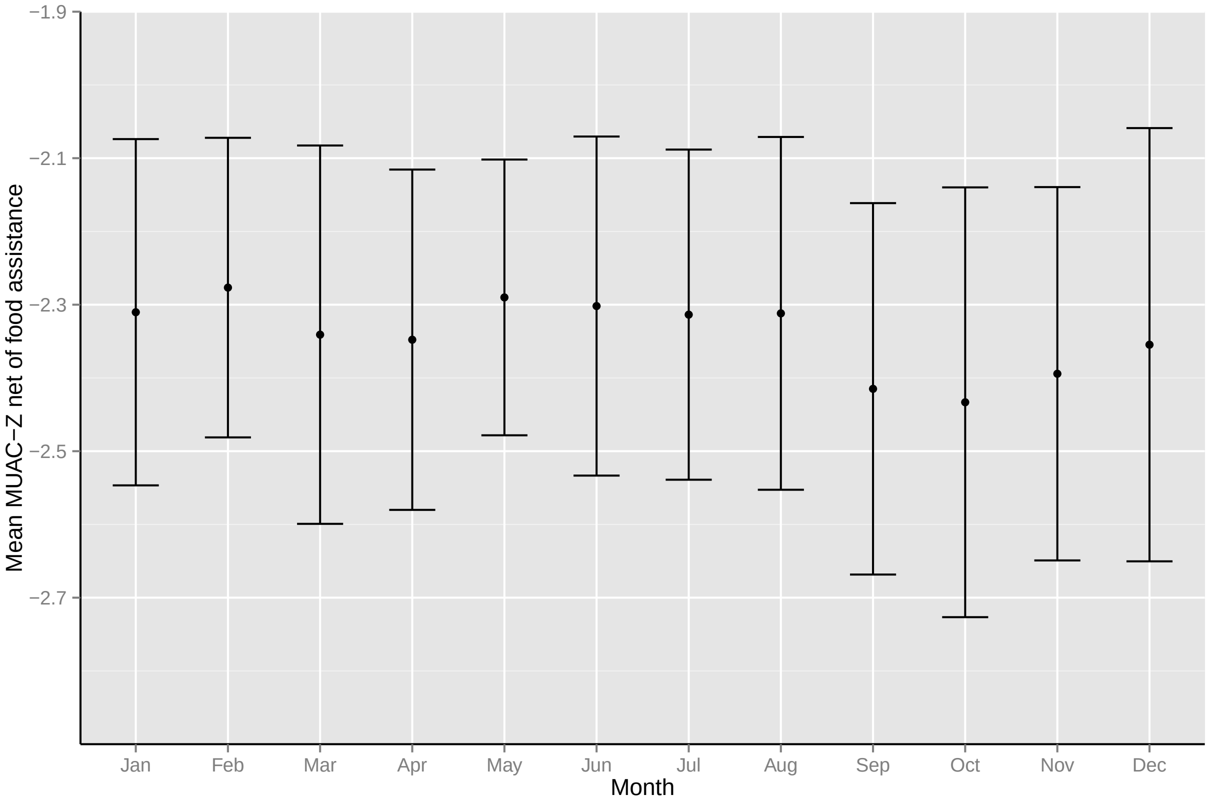

Fig B: The long-term mean MUAC-Z in the absence of food assistance for each month of the year, $\tilde{\mu}_{t \bmod 12}$.

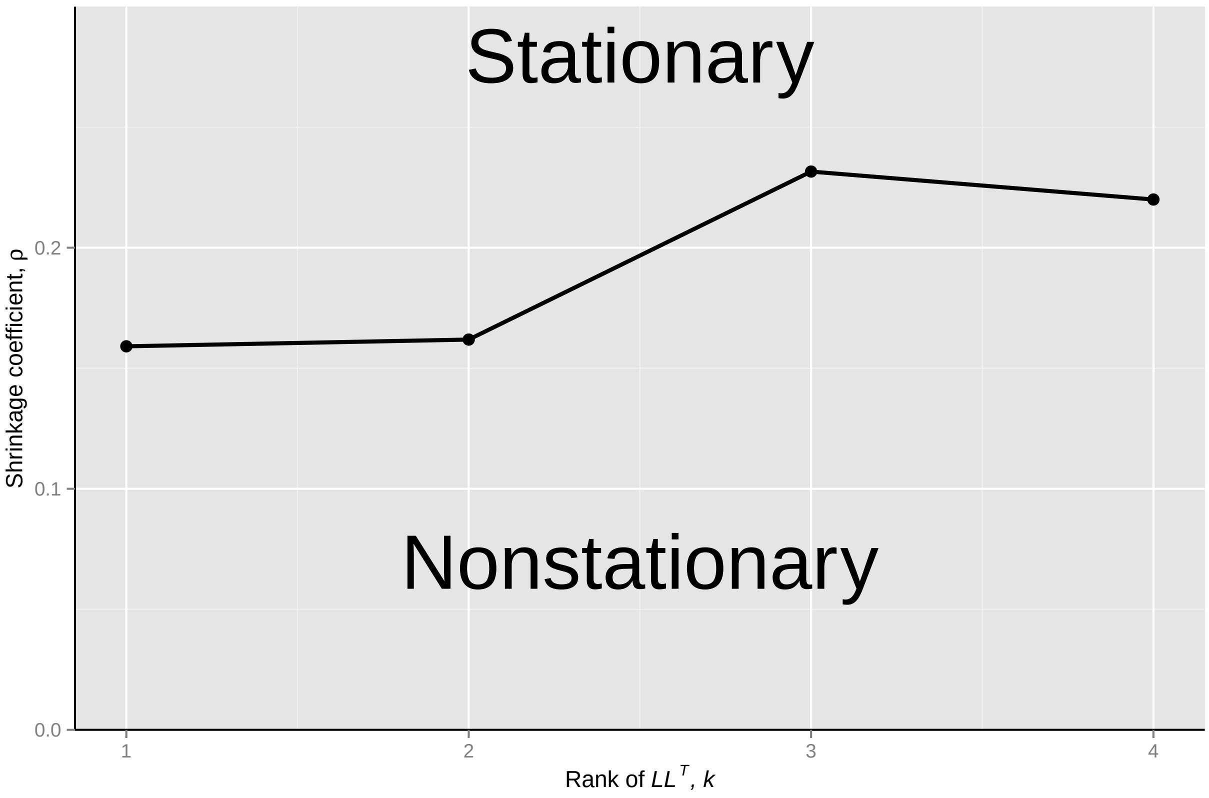

Fig C: Boundary between stationary and nonstationary estimates.

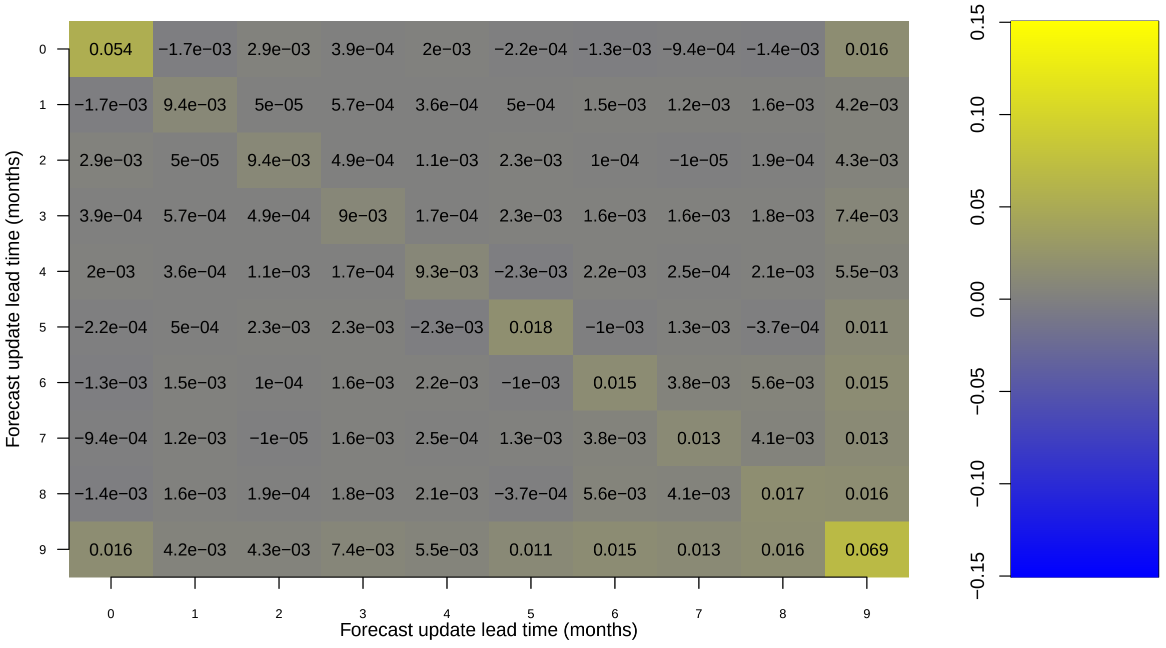

Fig D: The estimate of Σ^1.

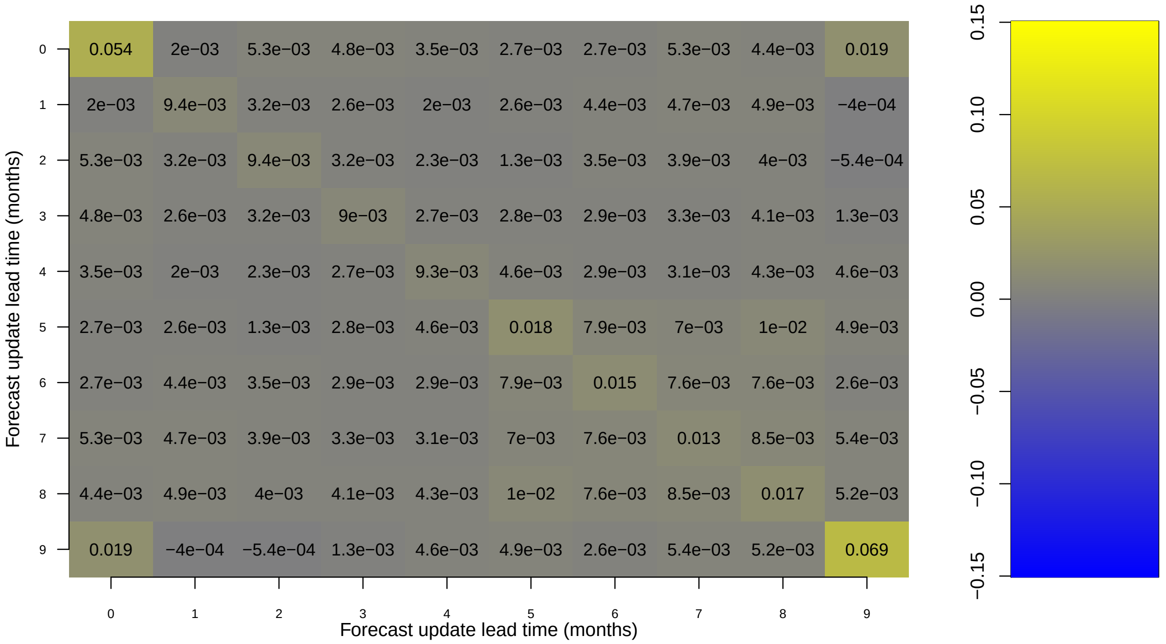

Fig E: The estimate of Σ^2.

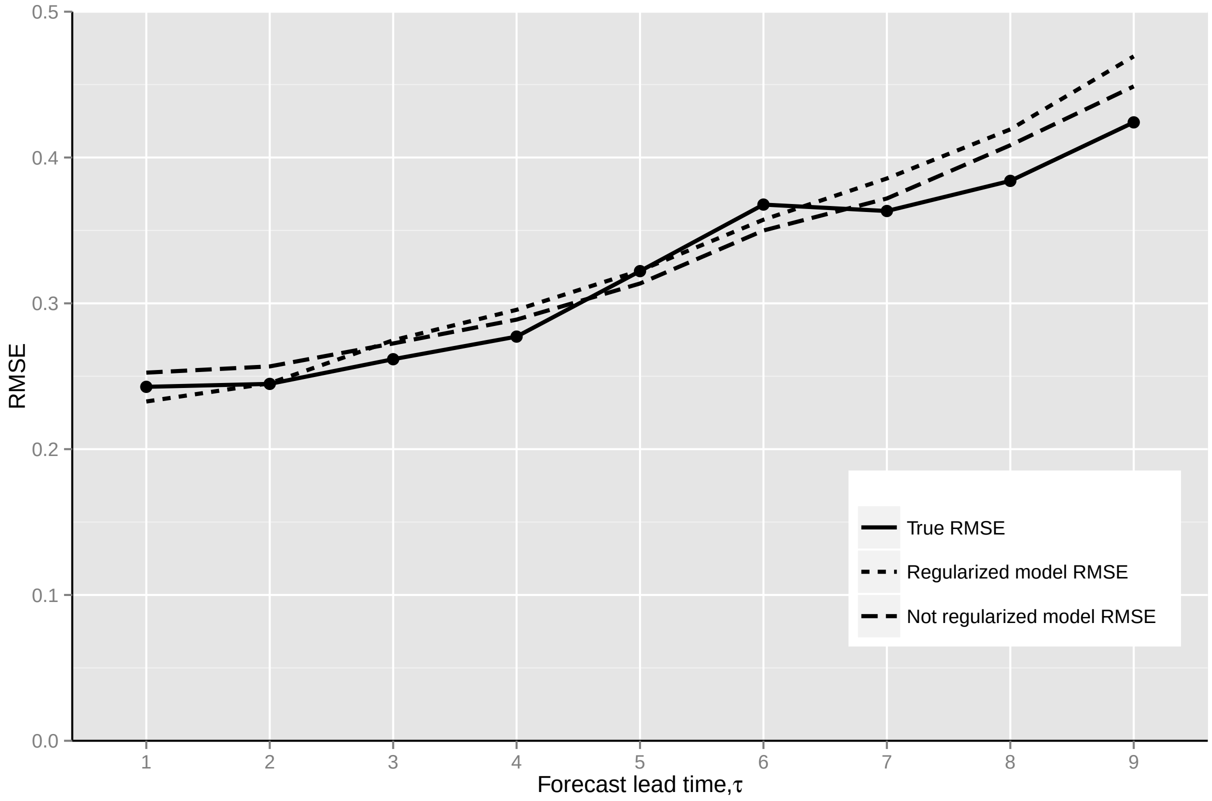

Fig F: A comparison of the true root mean square error (RMSE) and the model RMSE (with and without regularization).

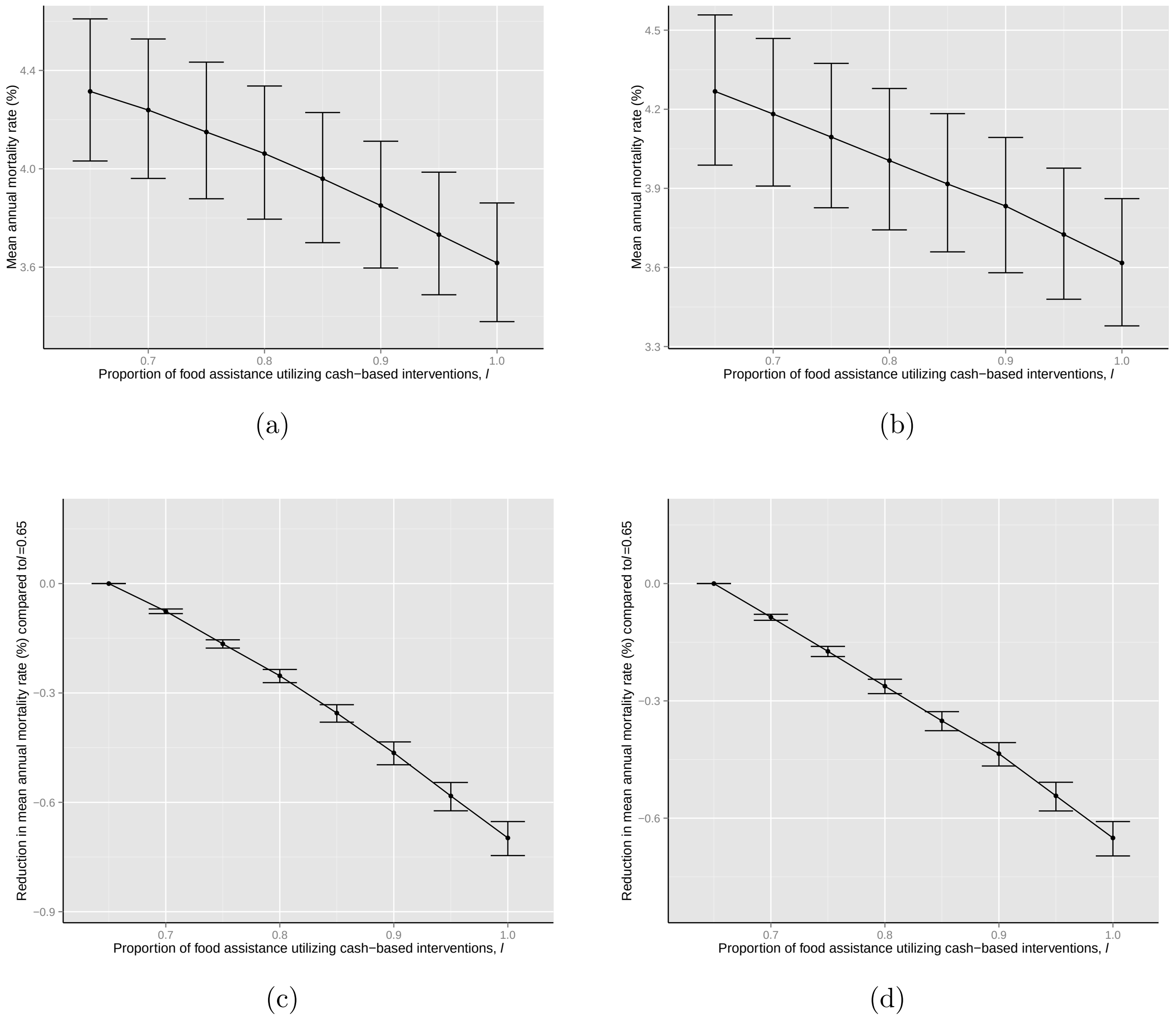

Fig G: 95% confidence intervals, which account for uncertainty in the two covariance matrices, the month-specific means of MUAC-Z and the variance of MUAC-Z in the population, for the mean annual mortality rate when **(a)** $p = 0.5$ and **(b)** $p = 1.0$, and for the absolute reduction in the mean annual mortality rate relative to the $l = 0.65$ case when **(c)** $p = 0.5$ and **(d)** $p = 1.0$.

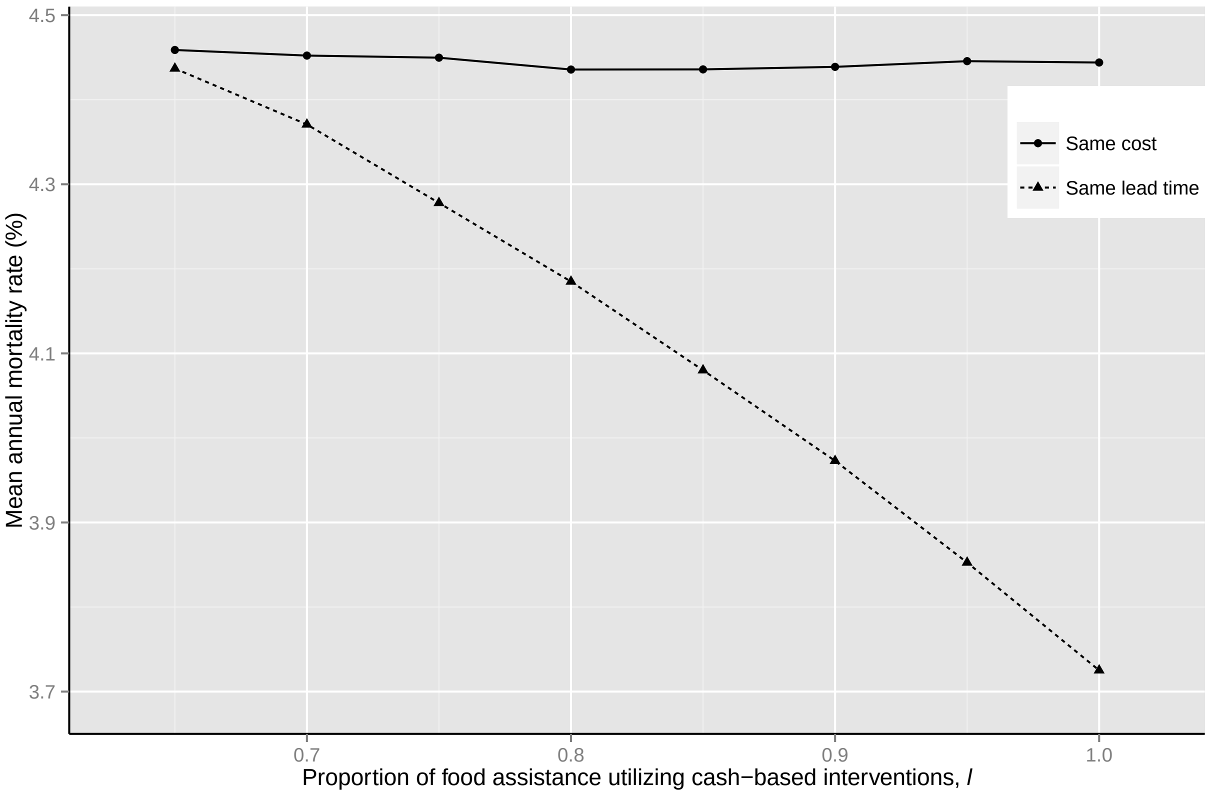

Fig H: Isolating the impacts of cost and lead times. For the cargo preference parameter $p = 0.5$, the dependence of the annual mortality rate on the proportion of food assistance utilizing case-based interventions (l), under the assumption (dashed line) that both procurement modes have the same lead time ($L_f = L_s = 3$ mo), and under the assumption (solid line) that both procurement modes have the same cost ($c_f = c_s =$\$0.506/kg).

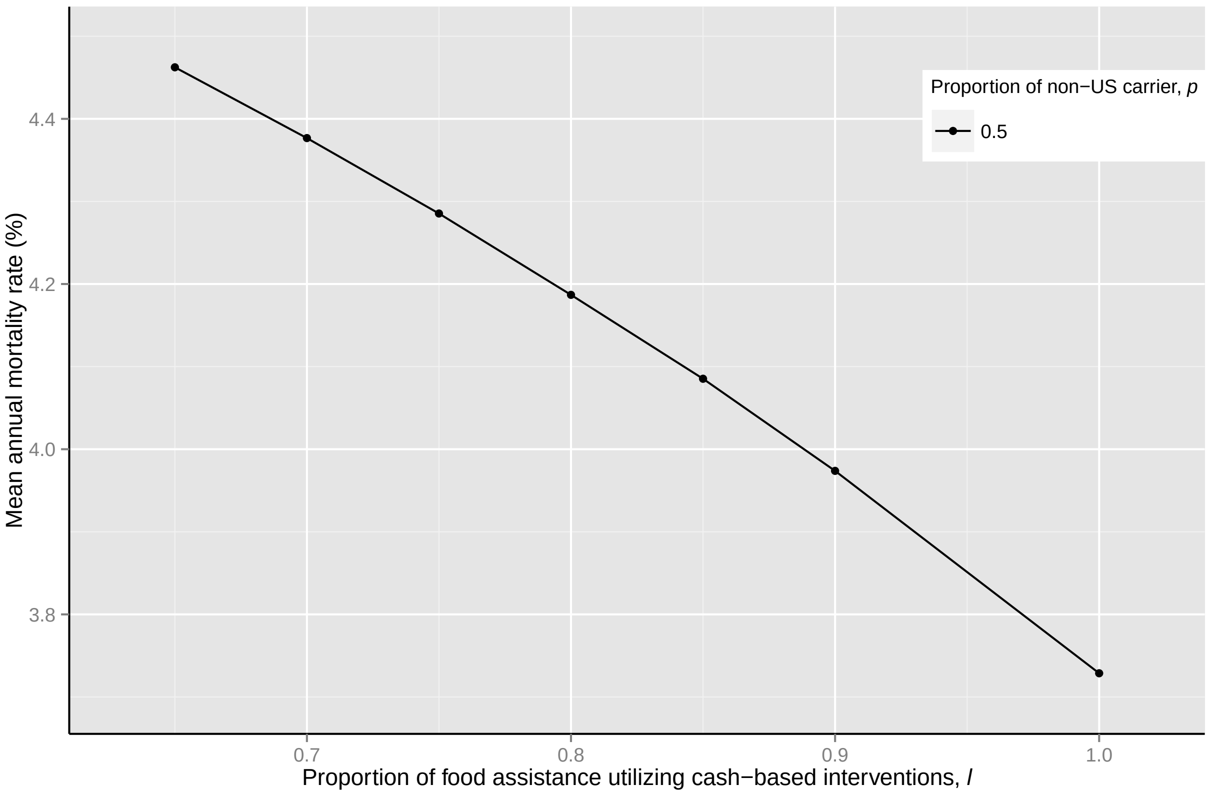

Fig I: Sensitivity analysis with respect to lead times. For the cargo preference parameter $p = 0.5$ and the generous budget ($B = \$19,867$), the dependence of the annual mortality rate on the proportion of food assistance utilizing case-based interventions (l), under the assumption that $L_f = 2$ mo and $L_s = 5$ mo.

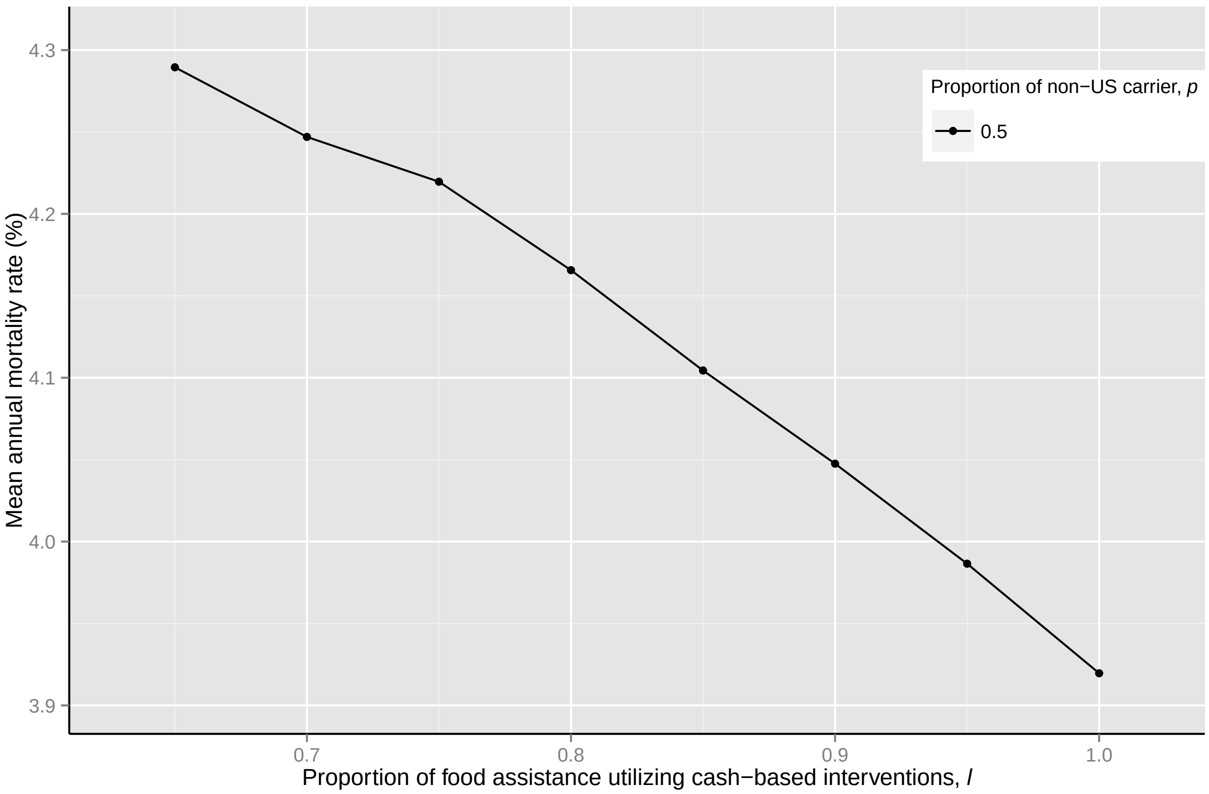

Fig J: Sensitivity analysis with respect to the food effect, d. For the cargo preference parameter $p = 0.5$ and the generous budget ($B = \$19,867$), the dependence of the annual mortality rate on the proportion of food assistance utilizing case-based interventions (l), under the assumption that $d = 0.0075/\text{kg}$.

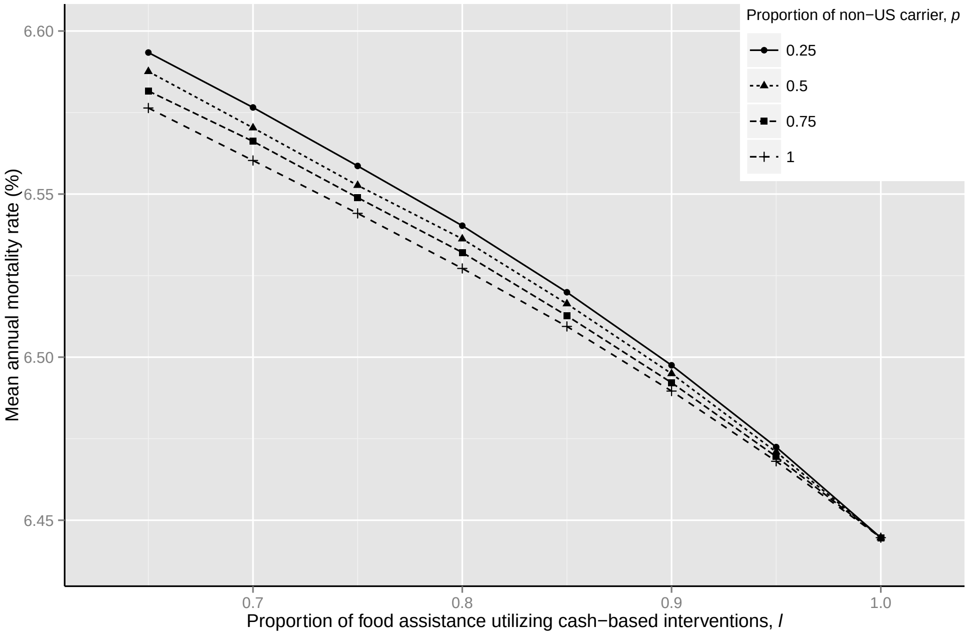

Fig K: Dependence of the annual mortality rate on the proportion of food aid utilizing cash-based interventions (l) and the proportion of transoceanic shipments employing non-US-flag carriers (p), under the restrictive budget ($B = \$2382$) considered in the sensitivity analysis.
